# Loss of laminin-γ1 in pericytes impairs microvascular function and cognition

**DOI:** 10.1016/j.celrep.2026.117904

**Published:** 2026-08-27

**Authors:** Jingsong Ruan, Ava Nasrollahi, Cecilia Martin, Meisam Asgari, Andy Y. Shih, Yao Yao

**Affiliations:** 1 Department of Molecular Pharmacology and Physiology, Morsani College of Medicine, University of South Florida, Tampa, FL, USA; 2 Center for Developmental Biology and Regenerative Medicine, Seattle Children’s Research Institute, Seattle, WA, USA; 3 Department of Medical Engineering, University of South Florida, Tampa, FL, USA; 4 Department of Pediatrics, University of Washington, Seattle, WA, USA; 5 Department of Bioengineering, University of Washington, Seattle, WA, USA; 6 Lead contact

## Abstract

Blood-brain barrier (BBB) disruption correlates with lesion burden and cognitive dysfunction in cerebral small vessel disease (CSVD). Laminin, highly produced by pericytes in the brain, maintains BBB integrity. However, it remains unclear if laminin dysregulation contributes to the pathogenesis of CSVD. Here, we show that mice lacking laminin-γ1 in pericytes, smooth muscle cells, and fibroblasts (PKO) develop age-dependent BBB disruption via both paracellular and transcellular mechanisms. In contrast, loss of laminin-γ1 in smooth muscle cells or fibroblasts fails to affect BBB integrity. In addition, the PKO mice exhibit pericyte loss, thickened basal lamina, increased brain stiffness, followed by brain clearance defect, synaptic loss, and cognitive impairment. Together, these results demonstrate that loss of pericytic laminin-γ1 induces progressive vascular dysfunction and several features of CSVD, including brain stiffness, synaptic loss, and cognitive impairment, highlighting a potential role of laminin-γ1 dysregulation in the pathogenesis of CSVD.

## In brief

Ruan et al. show that loss of pericytic laminin-γ1 induces vascular dysfunction and matrix defects at middle age, followed by CSF influx/brain clearance defects, synaptic loss, and cognitive decline at old age. These findings establish an important role of laminin dysregulation in the pathogenesis of cerebral small vessel disease.

## Graphical Abstract

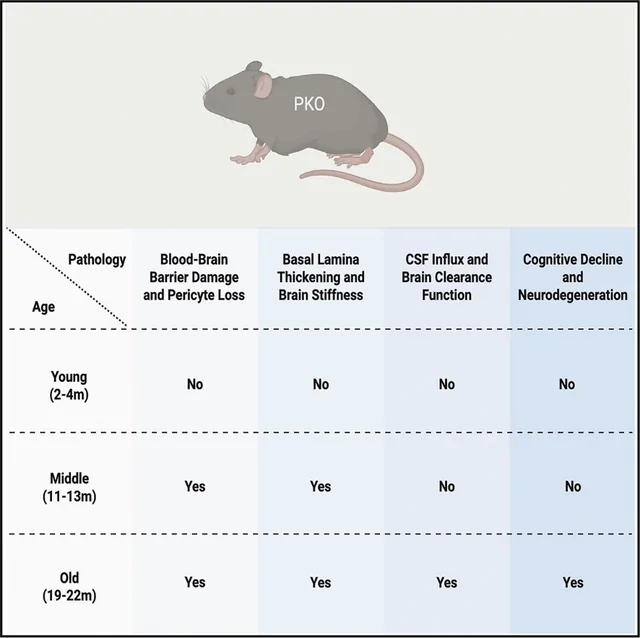

## INTRODUCTION

Cerebral small vessel disease (CSVD) refers to a heterogeneous collection of diseases related to aging, genetics, and hypertension that involve damage to the small vessels in the brain (arterioles, capillaries, and venules), causing vascular dysfunction, brain injury, and cognitive decline. CSVD affects 11 million people in the US and accounts for 20% of stroke and 45% of vascular dementia cases.^1,2^ Generally, CSVD pathology includes blood-brain barrier (BBB) disruption,^3–5^ loss of perivascular mural cells (pericytes and smooth muscle cells),^6^ increased tissue stiffness,^7,8^ and cerebral amyloid angiopathy (CAA)—the abnormal accumulation of amyloid-β (Aβ) in the basal lamina (BL) along vessels.

Correlation studies have shown that abnormal levels of BL components, including laminin and collagen IV, are associated with CAA pathology in both rodent models^9,10^ and postmortem human brains.^11^ In addition, mutations in BL genes, such as *COL4A1* and *LAMB1*, are involved in monogenic forms of CSVD.^12^ The role of vascular BL dysregulation in CSVD, however, remains poorly defined and needs further research. In this study, we focus on laminin, a major component of the BL.

Laminin is a heterotrimeric protein containing α, β, and γ subunits.^13,14^ In mammals, there are five α, four β, and three γ genetic variants, which generate a variety of laminin isoforms with potentially different functions.^15,16^ Among the three γ subunits, γ1 is the most frequently used one and has the widest expression.^17,18^ In the CNS, *LAMC1* (encoding laminin-γ1) is highly expressed in mural cells and fibroblasts, with the highest expression in pericytes, although other cells also express this gene.^19^ Consistent with its broad expression, global ablation of *Lamc1* leads to embryonic lethality at embryonic day 5.5 (E5.5),^20,21^ preventing investigation of its function at later stages. To overcome this issue, we generated conditional knockout (PKO) mice with *Lamc1* deficiency in pericytes, smooth muscle cells, and fibroblasts. PKO mice, rather than mutants with *Lamc1* deficiency in smooth muscle cells or fibroblasts, develop progressive age-dependent BBB disruption, indicating an essential role of pericytic laminin-γ1 in BBB integrity. In addition, PKO mice also exhibit pericyte loss, thickened BL, increased brain stiffness, defects in brain clearance of macromolecules, synaptic defects, and impaired cognitive function, recapitulating several pathologies of CSVD. Our results support a potential role of laminin dysregulation in the pathogenesis of CSVD.

## RESULTS

### PKO mice exhibit age-dependent BBB disruption

*Lamc1*^flox/flox^ mice were crossed with the *Pdgfrb*-Cre transgenic line to generate laminin-γ1 conditional knockout mice (*Lamc1*^flox/flox^:*Pdgfrb*-Cre^+^, termed PKO).^22–24^ Lineage-tracing studies confirmed that *Pdgfrb*-Cre induced recombination in pericytes, smooth muscle cells, and fibroblasts.^22,23^ Thus, laminin-γ1 is ablated in all three populations in PKO mice.

In our previous study,^25^ we showed that PKO mice had intact BBB integrity at 2–4 months but exhibited mild BBB breakdown at ~12 months, as evidenced by a 2-fold increase of fluorescein isothiocyanate (FITC)-dextran (4 kDa). Here, we further assessed BBB integrity in PKO at 19–22 months by immunohistochemistry (IHC) against hemoglobin and albumin, two blood proteins. Hemoglobin (64.5 kDa) was detected outside CD31^+^ blood vessels in the cortex of PKO but not control mice (Figure 1A). Quantifications revealed an over 3.5-fold increase of hemoglobin levels in both cortex (Figure 1B) and striatum (Figure 1C) of PKO mice compared to age-matched controls. Similarly, significantly higher (over 3-fold) levels of albumin (66.5 kDa) were found in the cortex and striatum of PKO mice compared to age-matched controls (Figures 1D–1F). The more dramatic (>3-fold) leakage of larger tracers (hemoglobin and albumin) at 19–22 months indicates exacerbated BBB disruption. Together, these results demonstrate that PKO mice develop age-dependent BBB disruption, highlighting an essential role of mural cell/fibroblast-derived laminin-γ1 in BBB maintenance.

To determine the role of fibroblast-derived laminin-γ1 in BBB integrity, we also generated mutant mice with laminin-γ1 deficiency in fibroblasts (*Lamc1*^flox/flox^:*Col1a1*-Cre^+^, termed FKO). Lineage tracing studies showed that *Col1a1*-Cre labels perivascular and meningeal fibroblasts specifically.^23,26^ Compared to age-matched controls, 19-month-old FKO mice exhibited similar levels of hemoglobin and FITC-dextran (4 kDa) leakage in both cortex and striatum (Figures S1A–S1F). Echoing these findings, control and FKO mice demonstrated similar levels of Mfsd2a and caveolin-1, transcytosis-associated proteins that tightly regulate BBB integrity, in both cortex and striatum (Figures S1G–S1L). These results indicate a minimal role of fibroblast-derived laminin-γ1 in BBB maintenance. Given that deletion of laminin-γ1 in smooth muscle cells fails to induce BBB disruption,^27^ BBB disruption in PKO mice is likely due to loss of laminin-γ1 in pericytes of the capillary bed, where the BBB predominantly resides. However, we cannot exclude the possibility that BBB disruption occurs only when laminin-γ1 from all three sources is depleted.

### Both paracellular and transcellular mechanisms contribute to BBB disruption in PKO mice

Brain endothelial cells form highly specialized tight junctions, marked by the expression of tight junction proteins (TJPs), including Claudin-5 and ZO-1. These TJPs seal gaps between adjacent endothelial cells, limiting paracellular leakage.^28^ In our previous study,^25^ we reported increased paracellular leakage in PKO mice at middle age. To determine if this mechanism is affected in PKO mice at old age, we examined TJP expression in control and PKO mice at 19–22 months by IHC. Claudin-5 co-localized with vascular marker podocalyxin in the cortex of both control and PKO mice (Figure 2A). Quantifications, however, revealed reduced Claudin-5 coverage in both the cortex (Figure 2B) and striatum (Figure 2C) of PKO mice compared to age-matched controls, although statistical significance was only achieved in the cortex (Figure 2C). Like Claudin-5, ZO-1 coverage was significantly reduced in the cortex and striatum of PKO mice compared to age-matched controls (Figures 2D–2F). These results suggest that TJP expression is decreased in PKO mice at old age. Consistent with these TJP changes, disrupted tight junctions (red arrowheads, Figure 2G) were frequently observed in 19- to 22-month-old PKO mice under transmission electron microscopy (TEM), indicating structural defects of the tight junctions. Together, these findings indicate that increased paracellular leakage contributes to BBB disruption in aged PKO mice.

Another key feature of brain endothelial cells is their extremely low rate of transcytosis, which maintains the transcellular barrier.^29^ In our previous study,^25^ we found enhanced endothelial transcytosis in PKO mice at middle age. To determine if this mechanism is affected in PKO mice at old age, we examined the expression of two transcytosis-associated proteins: Mfsd2a, a negative regulator of transcellular transport^30^; and Caveolin-1, a structural protein for caveolae and essential regulator of caveolae-mediated transcytosis.^31^ Mfsd2a co-localized with vascular marker podocalyxin in the cortex of both control and PKO mice (Figure 3A). Quantifications, however, revealed significantly lower Mfsd2a levels in both cortex (Figure 3B) and striatum (Figure 3C) of PKO mice compared to age-matched controls. Consistent with Mfsd2a’s role in regulation of transcytosis,^30,32^ caveolin-1 levels were significantly increased in the cortex (Figures 3D and 3E) and striatum (Figure 3F) of PKO mice compared to age-matched controls. At the ultrastructural level, endothelial pinocytotic vesicles were detected in the cortex of PKO mice at 19–22 months (arrowheads, Figure 3G). Quantification revealed significantly increased endothelial pinocytotic vesicle density in PKO brains compared to age-matched controls (Figure 3H). Subcellular distribution analysis revealed significantly increased pinocytotic vesicle density in the cytosol and abluminal membrane rather than luminal membrane of endothelial cells in PKO mice (Figures 3I–3K). Together, these findings indicate that increased transcellular leakage also contributes to BBB disruption in aged PKO mice.

### PKO mice show pericyte defects

Accumulating evidence indicates an important role of pericytes in BBB integrity.^33–35^ Our previous study showed that pericyte defects were detected in PKO mice at ~12 months but not 2–4 months,^25^ which correlated with BBB permeability. Here, we further characterized pericyte defects in PKO mice at 19–22 months. IHC showed diminished pericyte density in the cortex of PKO mice (Figure 4A). Quantifications revealed a 25%–35% reduction of pericyte coverage in both cortex (Figure 4B) and striatum (Figure 4C) of PKO mice compared to age-matched controls.

This loss of pericyte coverage was also observed *in vivo* using two-photon microscopy of PKO mice expressing tdTomato in PDGFRβ^+^ cells. Since pericytes arrange in a chain-like network along capillaries, loss of pericytes was evident as stretches of capillary endothelium lacking contact with any pericyte, while other regions remained in contact (Figure 4D). Analysis of pericyte cell body density (cells/mm of capillary length) revealed a decrease in PKO mice (Figure 4E). Importantly, there was no change in vascular density (Figure 4F). Given the relatively small cortical volume imaged by two-photon microscopy, we further analyzed vessel density by IHC on brain sections and found comparable vessel area and length in control and PKO mice (Figure S2). These findings suggest that pericyte loss, rather than increased vascular density, was driving the decrease in pericyte density. Together, these results highlight an essential role of pericytic laminin-γ1 in pericyte density and coverage over capillaries.

### PKO mice show BL thickening

The BL is an amorphous structure located between endothelial cells and astrocyte endfeet.^13,36,37^ Pericytes are embedded in the BL and separate it into two layers: endothelial BL (eBL) and parenchymal BL (pBL).^13,36,37^ The BL, eBL, and pBL were detected in the brains of both control and PKO mice at 19–22 months under TEM (Figure 4G). Quantifications, however, revealed significantly increased thickness of BL (Figure 4H), eBL (Figure 4I), and pBL (Figure 4J) in PKO brains compared to age-matched controls. This led to greater physical separation between endothelial cells and pericytes. Similarly, BL thickening was observed in PKO mice at ~12 months but not 2–4 months.^25^

To determine if compensatory deposition of other extracellular matrix (ECM) proteins may contribute to BL thickening in PKO mice, we examined the expression of collagen IV and nidogen1 at 11–13 months. Compared to the controls, PKO mice exhibited a higher level of collagen IV (Figures 4K and 4L). Interestingly, nidogen1 levels showed an increasing trend in PKO brains, although it did not reach statistical significance (Figures S3A and S3B). Together, these results suggest that pericytic laminin-γ1 contributes to the maintenance of BL structure, and its loss leads to BL thickening possibly via up-regulation of collagen IV.

### PKO mice show increased brain stiffness

Increased brain stiffness as a consequence of damage to the small blood vessels has been reported in CSVD.^7,8,38,39^ The increased brain stiffness mediates further damage, contributing to cerebrovascular dysfunction, brain atrophy, and cognitive impairment/dementia.^8,40,41^ To determine whether loss of pericytic laminin-γ1 affects brain stiffness, we measured the elastic modulus of brain tissues from control and PKO mice using atomic force microscopy (AFM). AFM vertical deflection and height images revealed distinct ultrastructural alterations in control and PKO brains at 11–13 months (Figure 5A). PKO brain tissue exhibited greater local variability in surface height, as evident from the standard deviation height maps (Figure 5B). Despite these local variations, no substantial overall differences in mean height across the tissue microstructure were observed between PKO and control brains. In contrast, brain stiffness showed pronounced differences. Local stiffness maps demonstrated a 3-fold increase in the maximum local elastic modulus in PKO brains compared to the controls (Figure 5C). Quantitative analysis across multiple samples and regions confirmed a statistically significant elevation in local elastic modulus in PKO brains (14.13 ± 8.46 kPa) compared to the controls (2.48 ± 0.77 kPa) (Figure 5D). These results strongly suggest that loss of pericytic laminin-γ1 increases brain stiffness.

One common cause of increased brain stiffness is ECM changes, which alter the cross-linking of ECM networks and the structure of the BL, leading to abnormal mechanical signaling and cell movement.^42,43^ Due to its intrinsic structural properties, collagen has been shown to render tissue stiffness.^44^ Consistent with the finding that collagen deposition is associated with increased vessel stiffness in atherosclerosis,^45^ we observed increased collagen IV expression in the capillary walls of PKO brains (Figures 4K and 4L), suggesting that enhanced collagen IV deposition may contribute to the elevated brain stiffness. In addition, we also examined reactive astrogliosis, which may also affect brain stiffness. IHC against GFAP revealed comparable astrocyte morphology in control and PKO mice at 11–13 months (Figure S3C). Quantification revealed similar GFAP levels in control and PKO mice at 11–13 months (Figure S3D), suggesting that the increased brain stiffness in PKO mice is unlikely due to reactive astrogliosis.

### PKO mice display compromised brain influx/efflux functions

Increased brain stiffness may affect the poroelasticity of brain tissue and increase resistance to fluid movement, thereby affecting cerebrospinal fluid (CSF) influx and the clearance of solutes from the brain.^46^ Further, in CAA, vascular stiffness is highly correlated with the pathological accumulation of Aβ in vascular BL.^47^ Specifically, vessels with Aβ aggregation have thicker BLs and are >300% stiffer than those without.^47^ To investigate whether solute clearance is affected by ablation of pericytic laminin-γ1, we intracerebrally injected Alexa Fluor 555-conjugated albumin (Albumin-555) into control and PKO mice at 19–22 months. Albumin-555 was detected in the brains of both control and PKO mice 1 h later (Figure 6A). Quantification, however, revealed a significantly higher level of Albumin-555 in the brains of PKO mice compared to age-matched controls (Figure 6B). A similar result was observed when HiLyte Fluor 647-labeled Aβ1–42 was used (Figure 6C), indicating reduced solute clearance in aged PKO mice. Interestingly, although brain stiffness was altered in PKO mice at 11–13 months (Figure 5), no differences in albumin or Aβ clearance were observed at this age (Figures S4A and S4B), indicating age-dependent deterioration of solute clearance.

CSF influx along perivascular spaces has been hypothesized to play a role in the clearance of Aβ along penetrating arterioles.^48^ As such, we examined this function in PKO mice by injecting Albumin-555 into the CSF via the cisterna magna. Albumin-555 was detected in the brain parenchyma of 19- to 22-month-old control and PKO mice 2 h later (Figure 6D). Quantification, however, showed substantially reduced Albumin-555 levels in the brains of aged PKO mice (Figure 6E). Similarly, a lower Alexa Fluor 647-conjugated IgG fraction was found in the brains of aged PKO mice (Figure 6F), suggesting diminished brain influx function in aged PKO mice. Again, no differences in albumin or IgG influx were observed in control and PKO mice at 11–13 months (Figures S4C and S4D), indicating age-dependent decline of perivascular fluid influx. Together, these findings highlight an important role of pericytic laminin-γ1 in CSF influx and brain clearance.

### PKO mice develop cognitive impairment and synaptic loss

BBB disruption, pericyte defects, BL thickening, abnormal brain stiffness, and compromised brain clearance are pathological features of CSVD, where the primary clinical outcome is cognitive impairment.^49–51^ To determine if PKO mice also develop cognitive impairment, we assessed the cognitive function of PKO mice using Morris water maze and Y maze tests. At 19–22 months, PKO mice exhibited substantially reduced time in the target quadrant with comparable total swimming distance compared to age-matched controls (Figures 7A–7C), suggesting cognitive impairment. The cognitive decline was detected in both male and female PKO mice (Figures S5A and S5B). Similar results were observed in the Y maze test. Specifically, old PKO mice showed decreased spontaneous alternation with comparable total arm entrances compared to age-matched controls (Figures 7D and 7E), again indicating cognitive deficits. Interestingly, this defect was mainly detected in female but not male PKO mice (Figures S5C and S5D).

Echoing cognitive decline, synaptic loss was observed in PKO mice at 19–22 months. Specifically, pre-synaptic marker synaptophysin was significantly reduced in the cortex of PKO mice compared to the controls (Figure 7F). Post-synaptic marker PSD95 was slightly decreased in the cortex of PKO mice, although statistical significance was not achieved (Figure 7G). Both synaptophysin and PSD95 showed significant reduction in the striatum of PKO mice compared to the controls (Figures 7H and 7I). These results suggest that PKO mice develop cognitive impairment and synaptic loss at 19–22 months.

In sharp contrast, control and PKO mice displayed comparable performance in both Morris water maze and Y maze tests at 11–13 months (Figures S6A–S6E). Similarly, no differences in synaptophysin or PSD95 expression were detected in control and PKO mice at this age (Figures S6F–S6I), suggesting intact cognitive function. These results suggest that PKO mice have not yet developed cognitive impairment or synaptic loss at 11–13 months. Together, these findings highlight a crucial and age-dependent role of pericytic laminin-γ1 in cognitive function and synapse health.

## DISCUSSION

In this study, we showed that loss of laminin-γ1 expression in pericytes led to vascular dysfunction (BBB disruption and reduced pericyte density/coverage) and matrix defects (BL thickening and increased brain stiffness) at 11–13 months, followed by compromised CSF influx and brain clearance, cognitive impairment, and synaptic loss at 19–22 months. These findings suggest that loss of pericytic laminin-γ1 is sufficient to drive microvascular dysfunction and cognitive impairment seen in CSVD in an age-related fashion.

The BBB is a dynamic structure that functions to maintain brain homeostasis. BBB disruption actively contributes to the pathogenesis of CSVD and neurodegenerative disorders, such as Alzheimer’s disease (AD). Located at the interface of the CNS and circulation system,^13,14^ laminin is well positioned to regulate BBB integrity. As a result, laminin dysregulation is associated with CSVD and AD. For example, a recent large-scale human genomics study found a strong correlation between *LAMC1* and hippocampal perivascular space burden,^52^ which is an early marker of CSVD.^53^ Abnormal laminin levels were also detected in CAA brains from both mice^9,10^ and humans.^11^ In addition, proteomics analysis revealed significantly decreased laminin-β1 levels in the CSF and brain parenchyma of patients with AD compared to age-matched controls,^54,55^ and a recent single-nucleus RNA sequencing analysis showed that pericytic expression of *LAMA4* and *LAMB1* was substantially reduced in the brains of patients with AD.^56^ These results suggest that laminin dysregulation may be an early event that contributes to the pathogenesis of these devastating disorders.

The BBB integrity is maintained by high levels of TJPs at the intercellular space and extremely low rates of transcytosis.^29,49^ Our findings demonstrated that loss of pericytic laminin-γ1 diminished TJP expression, impaired tight junction structure, and enhanced transcytosis, indicating the involvement of both paracellular and transcellular mechanisms. Thus, it is unlikely to reverse BBB disruption by blocking paracellular or transcellular transport alone. From a therapeutic point of view, targeting both pathways simultaneously will offer more beneficial effects.

Pericyte coverage, on a broad level across the capillary network, strongly correlates with BBB integrity.^33–35^ PKO mice exhibited significantly reduced pericyte coverage concurrent with BBB disruption. Compared to the controls, PKO mice also showed substantially thickened BL, eBL, and pBL. The thickening of BL may compromise cell-cell communications, including endothelial-pericyte crosstalk, which are important for pericyte survival.^57^ For example, it is likely that the thickened eBL may weaken PDGF-BB:PDGFRβ and/or TGF-β signaling pathways between endothelial cells and pericytes, and thus contribute to pericyte defects in PKO brains. However, this remains to be tested in future studies.

PKO mice displayed substantially increased brain stiffness, highlighting an important role of pericytic laminin-γ1 in tissue stiffness/elasticity. This is consistent with the reported function of the ECM in mechanical properties.^43,58^ Given that brain stiffness is elevated in CSVD,^7,8,38,39^ this finding suggests that loss of laminin-γ1 may contribute to this change in tissue property. Interestingly, abnormal brain stiffness has also been found in AD and frontotemporal dementia in a region-specific manner.^59^ In AD, brain stiffness is correlated with default mode network connectivity,^60^ and the medial temporal stiffness significantly predicts future cognitive decline.^61^ These findings suggest that laminin dysregulation may also be involved in the pathogenesis of AD.

In CAA, brain stiffness is highly correlated with the pathological accumulation of Aβ in vascular BL.^47^ Specifically, vessels with Aβ aggregation have thicker BLs and are >300% stiffer.^47^ Here, we show that the clearance of Aβ and albumin is affected by loss of pericytic laminin-γ1. The clearance of solutes from the brain can be attributed to a variety of mechanisms, including microglial and astrocyte uptake, BBB-mediated removal of Aβ via LRP-1, enzymatic degradation, changes in interstitial volume fraction, and perivascular clearance.^48,62,63^ Our data point us toward the latter two hypotheses. First, altered interstitial volume fraction across different sleep states drives changes in clearance.^62^ In PKO mice, the increased BL thickness observed on TEM could suggest an altered interstitial volume fraction contributing to the efflux defects. Second, loss of laminin-γ1 could also affect arteriole function, which is a driver of perivascular clearance.^64^ Perivascular clearance is based on the concept that waste removal occurs along spaces surrounding arterioles or venules in the brain, although the exact mechanism and direction of this waste removal are highly contested.^65^ Importantly, perivascular clearance can be influenced by physiological parameters such as blood pressure, heart rate, and breathing rate. We have shown that deletion of laminin-γ1 in smooth muscle cells leads to reduced blood pressure,^66^ although it remains unclear whether perivascular clearance is affected in these mutant mice. Nonetheless, these data open the door to further studies on whether laminin-γ1 plays a role in regulating local physiological dynamics of the microvasculature.

In summary, our study uncovers an important role of pericytic laminin-γ1 in cerebrovascular pathogenesis and vascular dementia. We demonstrate that PKO mice develop vascular dysfunction (BBB disruption and reduced pericyte density/coverage) and matrix defects (BL thickening and increased brain stiffness) at 11–13 months, and further exhibit CSF influx and brain clearance defects, cognitive impairment, and synaptic loss at 19–22 months. These findings suggest a potential role of laminin dysregulation in the pathogenesis of CSVD.

### Limitations of the study

This study has several limitations. First, different anesthesia methods were used for efflux assay (isoflurane) and influx assay (avertin). Although both control and PKO mice were tested under the same conditions within each assay, it is important to know that both isoflurane and avertin inhibit CSF tracer influx.^67^ It would be interesting to see if influx/efflux functions are affected in PKO mice with ketamine/xylazine anesthesia, which has the least effect on glymphatic dynamics.^67^ Second, PKO mice demonstrated reduced survival at 15–22 months compared to the controls (Figure S7). Most deceased mice had hemorrhages in their brains, suggesting that brain hemorrhages may be a possible cause of death. The higher mortality rate of PKO mice introduces a selection bias: the tested cohort represents survivors. These findings highlight critical roles of pericytic laminin-γ1 in vascular integrity and animal survival at old age. Third, although PKO and control mice showed comparable floating time and no mice were excluded from analysis, the Morris water maze induces higher stress than alternative cognitive tests, such as the Barnes maze,^68^ which disproportionately affects aged animals. Thus, care should be taken when interpreting the results.

## RESOURCE AVAILABILITY

### Lead contact

Further information and requests for resources and reagents should be directed to and will be fulfilled by the lead contact, Yao Yao (yao7@usf.edu).

### Materials availability

Materials will be shared upon request within the limits of the respective material transfer agreements.

### Data and code availability

- Raw data for Figures 1, 2, 3, 4, 5, 6, and 7; Figures S1–S7 were deposited on Digital Commons: http://www.doi.org/10.17632/t8hszf89km.2.
- This paper does not report original code.
- Any additional information required to reanalyze the data reported in this paper is available from the lead contact upon request.

## STAR★METHODS

### EXPERIMENTAL MODEL AND STUDY PARTICIPANT DETAILS

#### Mouse generation

*Lamc1*^flox/flox^ mice were crossed with the *Pdgfrb*-Cre transgenic line^22–24^ to generate PKO (*Lamc1*^flox/flox^:*Pdgfrb*-Cre^+^) mice. *Lamc1*^flox/flox^ mice were also crossed with the fibroblast-specific *Col1a1*-Cre line^19,26,69^ to generate FKO (*Lamc1*^flox/flox^: *Col1a1*-Cre^+^) mice. Wildtype littermates (*Lamc1*^flox/flox^) were used as controls. Mice were maintained in the C57BL6 dominant background. Both male and female mice at the middle age (11–13m) and old age (19–22m) were used in this study.

#### Mouse maintenance

All mice were maintained in the animal facility at the University of South Florida. They were kept in an environment with a temperature of 21°C–23°C, humidity of 30%–70%, and a 12h/12h (light/dark) cycle. They were kept in ventilated cages with free access to water and food, under specific pathogen-free conditions. For *in vivo* two-photon imaging experiments, PKO mice were shipped from University of South Florida to Seattle Children’s Research Institute. Control mice for *in vivo* imaging were age-matched PDGFRβ-tdTomato mice. All experimental procedures were in accordance with the NIH guide for care and use of animals and were approved by the Institutional Animal Care and Use Committee (IACUC) at the University of South Florida and Seattle Children’s Research Institute. Experiments were conducted and reported per the ARRIVE guidelines.

### METHOD DETAILS

#### Tissue collection

Mice were anesthetized and transcardially perfused with PBS followed by 4% paraformaldehyde. After perfusion, the brain was removed from the skull carefully and frozen on dry ice immediately. The frozen brains were then sectioned using a cryostat (CryoStar NX50, Thermo Fisher Scientific). Coronal serial sections, with a thickness of 20 μm, were collected throughout the whole brain for the following analysis.

#### Immunohistochemistry

Brain sections were fixed in 4% PFA for 20 min and washed in PBS 3 times. The sections were then incubated with IHC blocking buffer (1% BSA, 0.3% normal donkey serum, and 0.3% Triton X-100 in PBS) for 1 h, followed by overnight incubation with primary antibodies: rabbit anti-hemoglobin (1:400, Cloud-Clone, PAB409Mu01), goat anti-albumin (1:200, Novus Biologicals, NB600–41532), rabbit anti-claudin-5 (1:200, Thermo Fisher Scientific, 34–1600), rabbit anti-ZO-1 (1:400, Thermo Fisher Scientific, 61–7300), goat anti-podocalyxin (1:400, R&D systems, AF1556), rabbit anti-Mfsd2a (1:400, a kind gift from Dr. Chenghua Gu), mouse anti-caveolin-1 (1:400, Santa Cruz, sc-70516), rat anti-CD31 (1:100, BD, 553370), rabbit anti-collagen IV (1:500, Abcam, ab6586), rat anti-nidogen1 (1:500, Thermo Fisher Scientific, MA1–06501), rabbit anti-GFAP (1:400, Thermo Fisher Scientific, PA1–10019), and rat anti-PDGFRβ (1:200, eBioscience, 14–1402). The sections were washed in PBS 3 times and then incubated with appropriate fluorescent secondary antibodies for 1 h. The sections were then washed in PBS 3 times and mounted using fluoromount-G with DAPI.

For BBB leakage quantification, the intensity and/or area of hemoglobin, albumin, and FITC-dextran (4 kDa) were determined using ImageJ (NIH). BBB leakage in PKO mice was normalized to that of wildtype controls. For claudin-5 and ZO-1 coverage quantification, the areas of claudin-5, ZO-1, and podocalyxin were determined using ImageJ (NIH). Claudin-5 and ZO-1 coverage were calculated as the percentages of claudin-5 and ZO-1 areas over podocalyxin area, respectively. For Mfsd2a and Caveolin-1 intensity quantification, podocalyxin-positive vessel area was first selected by thresholding. The mean gray value of Mfsd2a and caveolin-1 within the selected area was measured using ImageJ (NIH). Three random images from each section, three to four coronal sections per brain, and four animals per group were used for quantifications.

#### Histological analysis of pericyte coverage

Pericyte coverage analysis was performed as previously described.^33^ Briefly, endothelial cells and pericytes were stained using podocalyxin and PDGFRβ antibodies, respectively. Pericyte coverage was quantified as the percentage (%) of PDGFRβ^+^ pericyte area covering podocalyxin^+^ capillary area. PDGFRβ^+^ and podocalyxin^+^ areas were measured using the ImageJ software (area measurement tool). Three random images from each section, three coronal sections per brain, and four animals per group were used for quantification.

#### Brain influx/efflux assays

Brain influx and efflux assays were performed as described previously.^70^ For influx assay, mice were anesthetized with 1.25% avertin. Although avertin is less ideal for perivascular influx study compared to other anesthetics,^67^ labeling was sufficient for a comparative analysis between genotypes. Next, the head was fixed on a stereotaxic frame (RWD Life Science Inc.) and slightly tilted so that it formed an angle of 120° to the body. A midline incision was made in the skin overlying the neck, and the neck muscles were separated carefully to expose the cisterna magna. 5 μL of Alexa Fluor 647-conjugated goat IgG (2 mg/mL) or Alexa Fluor 555-conjugated albumin (0.5 mg/mL) was injected into the cisterna magna compartment at the rate of 500 nL/min using a 10 μL Hamilton syringe (Hamilton Company). After injection, the syringe needle was left in place for an additional 3 min to prevent backflow. The incision was then closed, and the mouse was subcutaneously injected with buprenorphine (0.05 mg/kg) and allowed to recover on a heating pad. Mice were transcardially perfused 2 h after injection and the brains were cut into 100 μm sections using a Leica VT1000S vibratome (Leica). Serial sections throughout the whole brain were collected on one slide and all sections were imaged using a fluorescence BZ-X600 microscope (Keyence) under 2*x*. The areas of fluorescent signal and brain tissue in each section were determined. Influx fraction (%) was quantified as the percentage of fluorescent signal area over brain tissue area.

For efflux assay, mice were anesthetized with 1% isoflurane. 1 μL of Alexa Fluor 555-conjugated albumin (0.5 mg/mL, Thermo Fisher Scientific) or 2 μL of HiLyte Fluor 647-labeled Aβ 1–42 (0.05 mg/mL) was injected into the striatum (AP: +1.5 mm, ML: −1.5 mm, DV: −3.0 mm) at the rate of 400 nL/min using a 5 μL Hamilton syringe (Hamilton Company). After injection, the syringe needle was left in place for an additional 5 min to prevent backflow. Mice were transcardially perfused 1 h after injection and the brains were cut into 20 μm sections using a cryostat (CryoStar NX50, Thermo Fisher Scientific). Serial sections were collected and all sections containing fluorescent tracers were imaged using a fluorescence BZ-X600 microscope (Keyence) under 2*x*. The areas of fluorescent signal and brain tissue in each section were determined. Efflux fraction (%) was quantified as the percentage of fluorescent signal area over brain tissue area.

#### Thinned-skull cranial windows

Cranial windows were generated as described previously, with some adaptations.^71^ Briefly, surgical plane anesthesia was achieved with an isoflurane/oxygen mixture at 4% mean alveolar concentration (MAC) induction and ~2% MAC holding throughout surgery (Patterson Vet, 07–894-8943). Mice were provided oxygenation through medical air (20–22% oxygen and 78% nitrogen, moisturized by bubbling through water; AirGas Inc.). Prior to the incision mice were injected subcutaneously with 50 μg/kg buprenorphine for analgesia (Patterson Vet, 07–894-9214). Under sterile conditions, the scalp was excised and the periosteum cleaned from the skull surface. The incision borders were stabilized with Loctite Instant Adhesive 401 followed by C&B-Metabond quick adhesive cement (Parkell; S380). A custom aluminum head-bolt was attached to the right side of the skull surface using the C&B-Metabond quick adhesive cement (Parkell; S380). A sterile drill bit was used to thin the skull to ~20 μm across ~3 mm in diameter over the primary somatosensory cortex in the left hemisphere. A 3 mm glass coverslip (Warner Instruments, 64–0720) was adhered to the thinned skull area with Loctite Instant Adhesive 401. The remaining exposed skull was sealed with C&B-Metabond quick adhesive cement (Parkell; S380). Throughout surgery and during recovery from anesthesia, body temperature was maintained at 37°C with a feedback regulated heat pad (FHC Inc.). Post-procedure monitoring every 12 h for 48 h following surgery included pain management and hydration via subcutaneous 50 μg/kg buprenorphine and subcutaneous Ringer’s Solution (Thermo Fisher Scientific, AAJ67572AP). Imaging was initiated after a 48-h recovery period.

#### *In vivo* anesthetized two-photon imaging

Isoflurane (4% MAC in medical air) was used for induction of anesthesia and mice were head fixed to imaging apparatus. To visualize the vasculature, 100 μL of 5% (w/v in sterile saline) 2 MDa FITC-dextran was injected through the retro-orbital vein, where it is retained in the blood plasma to form a fluorescent “angiogram”.^72^ *In vivo* two-photon imaging was conducted using a Bruker Investigator microscope (run by Prairie View 5.5 software) coupled to a Spectra-Physics Insight *X*3 DA laser source (SpectraPhysics). Endogenous PDGFRβ-driven tdTomato fluorescence and Alexa 680-labeled microvasculature were imaged simultaneously at 1100 nm excitation and emission was collected through 525/70-nm and 700/75-nm bandpass filters. During imaging, laser power ranged between 10 and 100 mW, with higher powers only used briefly to visualize deeper tissues. Image analysis of pericyte cell density was performed in Imaris by counting cell somata. Image analysis of vascular density was performed in Imaris through vessel segmentation. All *in vivo* statistical analysis was done in GraphPad Prism via a Mann-Whitney test and plotted as median with 95% CI.

#### Transmission electron microscopy

TEM was performed as described in our previous publications.^26,73^ Briefly, mice were anesthetized and perfused with PBS followed by 0.1M sodium cacodylate buffer containing 2% paraformaldehyde and 2% glutaraldehyde. After perfusion, brain tissue was dissected out and fixed in 4% PFA with 0.1 M Na-cacodylate overnight. The tissue was then cut into 100 μm sections using a Leica VT1000 S vibratome, and post-fixed in 1% osmium tetroxide. Next, the sections were stained with 2% uranyl acetate and embedded in resin. The embedded tissue was then cut into ultra-thin sections using a PTPCZ PowerTome (RMC Boeckeler). Sections were examined and photographed under a TEM (JEOL JEM-1400Flash).

BL thickness measurement was performed in ImageJ using uniform capillary cross-sections that contain pericytes. BL thickness was determined in regions without pericytes by sampling along the entire lengths of BLs. eBL and pBL thickness were calculated by measuring the thickness of BL layer between endothelial cells and pericytes as well as that between pericytes and astrocyte endfeet, respectively. At least 15 measurements evenly distributed across the length of each pericyte were performed and averaged to calculate the mean thickness per capillary. For quantifications, 64–92 capillaries from 6 brains were used for quantifications.

Total and cytosolic transcytotic vesicle density was quantified by normalizing the numbers of transcytotic vesicles in all parts (cytosol and luminal/abluminal membranes) and cytosol of endothelial cells to the area of endothelial cells, respectively. Luminal and abluminal transcytotic vesicle density was quantified by normalizing the number of luminal and abluminal transcytotic vesicles in endothelial cells to the length of luminal and abluminal membranes of endothelial cells, respectively. 73–88 capillaries from 6 brains were used for quantifications.

#### Morris water maze

Morris water maze test was performed as described previously.^74^ Briefly, animals were trained for four days with 5 trials each day. During each trial, mice were allowed to find the hidden platform in the pool for 1 min. An intertrial interval of 2 min was used between each training. If mice failed to find the platform, they were guided to the platform and left there for 15 s. Mice were released to the pool from the same location on days 1 and 2, but from different locations on days 3 and 4 to establish a spatial cognition of the apparatus. On day 5, the platform was removed, and mice were allowed to explore the pool for 1 min. The time spent in each quadrant and total swimming distance were recorded. More time spent in the quadrant where the platform was before indicates better spatial recognition. Floating time, defined as the time of passive swimming at a velocity of <3 cm/s, was also calculated. Animals exhibiting floating time for >20 s in a single trial were excluded. Based on this criterion, no mice were excluded in this study.

#### Y maze test

The Y maze test was performed as described previously.^74^ Briefly, a three-arm Y maze was used as the apparatus and each arm was marked with different numbers. Mice were put into the starting arm marked as arm 1 and allowed to explore the Y-maze for 5 min. The sequence of entry to each arm was recorded. Spontaneous alternation is calculated as the percentage of consecutive entries into 3 different arms of the maze without any repeats in any order over total entrance. Higher spontaneous alternation indicates better working memory. Total arm entrance was also quantified to determine the overall exploratory motivation.

#### Atomic force microscopy

A JPK Nanowizard PURE AFM (Bruker Nano), mounted on an inverted Zeiss Axiovert 200 M epifluorescence microscope (Carl Zeiss Microscopy), was used to assess the mechanical properties of tissue sections. Imaging was performed using MSNL-10 silicon nitride cantilevers (Bruker), featuring a spring constant of 0.01–0.1 N/m and a 2-nm tip radius. For mechanical probing, Biosphere Au Reflex (CONT-Au) cantilevers (Nanotools USA LLC) were used, featuring a nominal spring constant of 0.2 N/m, a length of 450 μm, and a nominal resonance frequency of 13 kHz in air, with a spherical tip diameter of 10 μm (±10%).^75^ Tissue sections were prepared by frozen sectioning and mounted on microscope slides. Prior to testing, they were thawed following a previous well-established protocol.^75–77^ Light microscopy was used to precisely position the AFM probe over specific regions for indentation. All measurements were conducted in a hydrated state, with both the tissue and cantilever submerged in PBS. For each experimental condition, more than 2200 force curves were collected. Data were obtained from three pairs of slides (three pairs of control and PKO mice), with each slide containing three tissue sections that were tested. Within each section, measurements were taken from four to five distinct zones, with 50–100 force curves recorded per zone.

Before each experiment, the deflection sensitivity of the AFM cantilever was calibrated using a clean microscope slide.^77^ The precise spring constant was determined via thermal noise analysis in air, where the primary resonance peak was fitted to a simple harmonic oscillator model using JPK software.^77^ Indentation tests were performed at a rate of 2 μm/s. Since adhesion forces were negligible in this tissue, the elastic modulus (E) was calculated using the Hertz contact model applied to force-displacement curves.^75^ AFM data analysis was conducted using JPK data processing software.

#### Western blotting

Cortex and striatum tissues were homogenized in RIPA buffer using a Branson Ultrasonics Sonifier (Thermo Fisher Scientific). Protein concentration was determined using BCA Protein Assay Kit (Pierce, 23227). Equal amounts of proteins were separated in SDS-PAGE and transferred onto PVDF membranes. The membranes were blocked in 5% non-fat milk for 60 min, followed by incubation with anti-synaptophysin (Santa Cruz, SC-12737, 1:500), anti-PSD95 (Abcam, ab18258, 1:1000), and anti-β-actin (BD, 612657, 1:2000) overnight at 4°C. The membranes were washed and incubated with appropriate HRP-conjugated secondary antibodies for 60 min at room temperature the next day. The membranes were then washed and developed with Clarity Max^™^ Western ECL Substrate (Bio-Rad, 1705062), followed by imaging using the ChemiDoc XRS+ imaging system (Bio-Rad). The integrated density values of target proteins were obtained using ImageJ (NIH) and normalized to that of β-actin.

### QUANTIFICATION AND STATISTICAL ANALYSIS

All statistical analyses were performed using the GraphPad Prism 9 software (GraphPad Software). For normally distributed measurements, unpaired student’s *t* test (for two groups) and two-way ANOVA (for three or more groups) were used to determine statistical significance. For non-normally distributed measurements, Mann-Whitney test was used to determine the statistical significance. For survival rate, Log-rank test was used to determine the statistical significance. A *p*-value <0.05 was deemed to be statistically significant. All statistical details can be found in the figure legends. Brain stiffness data were shown using boxplots. For other measurements, data were shown as Mean ± SD.

## Supplementary Material

1

SUPPLEMENTAL INFORMATION

Supplemental information can be found online at https://doi.org/10.1016/j.celrep.2026.117904.

## Figures and Tables

**Figure 1.**
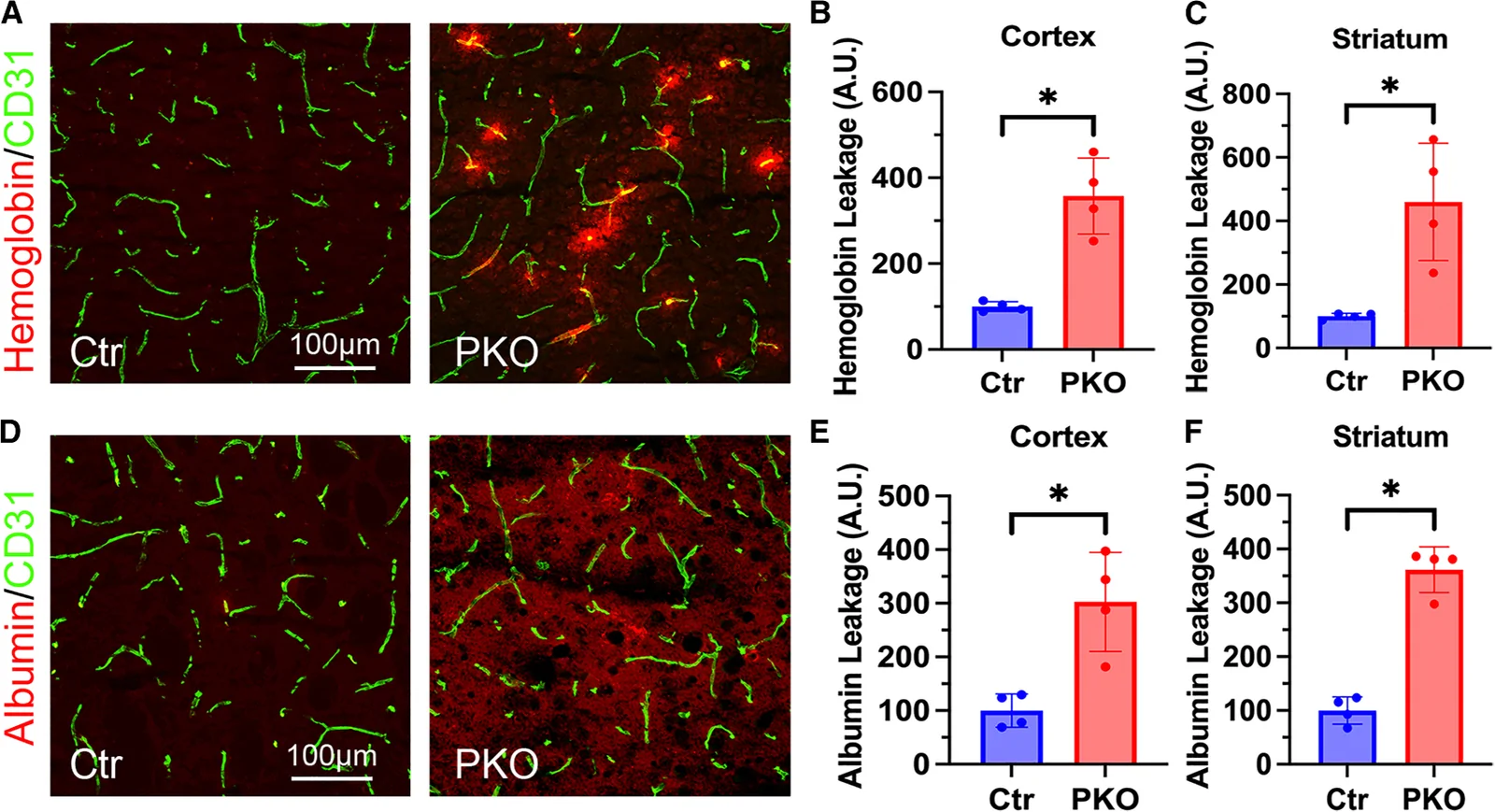
BBB integrity is disrupted in PKO mice at 19–22 months (A) Immunohistochemistry showing hemoglobin (red) and CD31 (green) staining in the cortex of 19- to 22-month-old control and PKO mice. Scale bars, 100 μm. (B) Quantification of hemoglobin leakage in the cortex of 19- to 22-month-old control and PKO mice. *n* = 4 mice. *p = 0.0286, Mann-Whitney test. (C) Quantification of hemoglobin leakage in the striatum of 19- to 22-month-old control and PKO mice. *n* = 4 mice. *p = 0.0286, Mann-Whitney test. (D) Immunohistochemistry showing albumin (red) and CD31 (green) staining in the cortex of 19- to 22-month-old control and PKO mice. Scale bars, 100 μm. (E) Quantification of albumin leakage in the cortex of 19- to 22-month-old control and PKO mice. *n* = 4 mice. *p = 0.0286, Mann-Whitney test. (F) Quantification of albumin leakage in the striatum of 19- to 22-month-old control and PKO mice. *n* = 4 mice. *p = 0.0286, Mann-Whitney test. Data were shown as mean ± SD.

**Figure 2.**
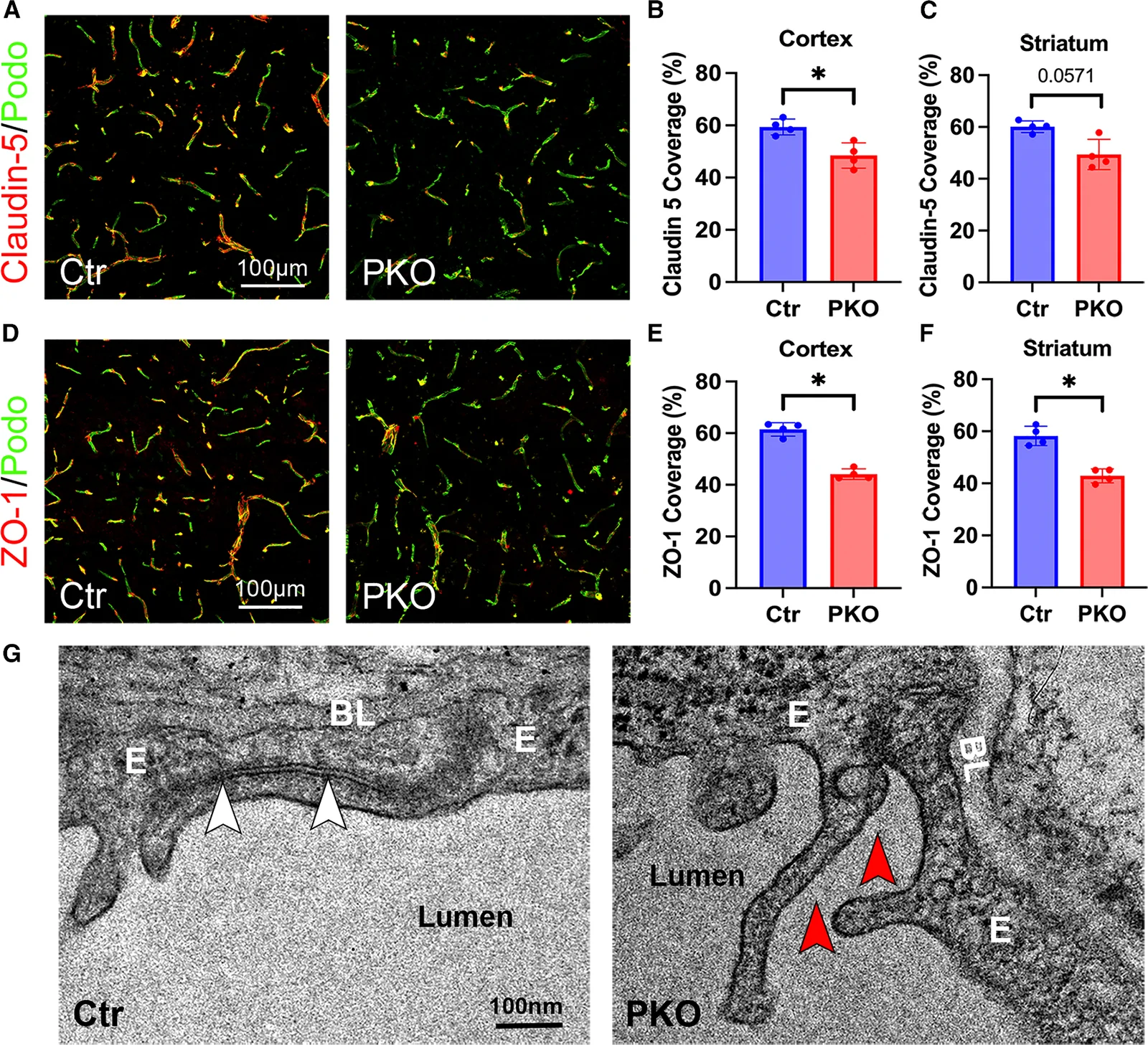
Paracellular transport is increased in PKO mice at 19–22 months (A) Immunohistochemistry showing claudin-5 (red) and podocalyxin (green) staining in the cortex of 19- to 22-month-old control and PKO mice. Scale bars, 100 μm. (B) Quantification of claudin-5 coverage in the cortex of 19- to 22-month-old control and PKO mice. *n* = 4 mice. *p = 0.0286, Mann-Whitney test. (C) Quantification of claudin-5 coverage in the striatum of 19- to 22-month-old control and PKO mice. *n* = 4 mice. ns, *p* = 0.0571, Mann-Whitney test. (D) Immunohistochemistry showing ZO-1 (red) and podocalyxin (green) staining in the cortex of 19- to 22-month-old control and PKO mice. Scale bars, 100 μm. (E) Quantification of ZO-1 coverage in the cortex of 19- to 22-month-old control and PKO mice. *n* = 4 mice. *p = 0.0286, Mann-Whitney test. (F) Quantification of ZO-1 coverage in the striatum of 19- to 22-month-old control and PKO mice. *n* = 4 mice. *p = 0.0286, Mann-Whitney test. (G) Representative TEM images showing intact tight junctions (white arrowheads) in control brains and disrupted tight junctions (red arrowheads) in PKO brains at 19–22 months. TEM, transmission electron microscopy; E, endothelium; BL, basal lamina. Data were shown as Mean ± SD.

**Figure 3.**
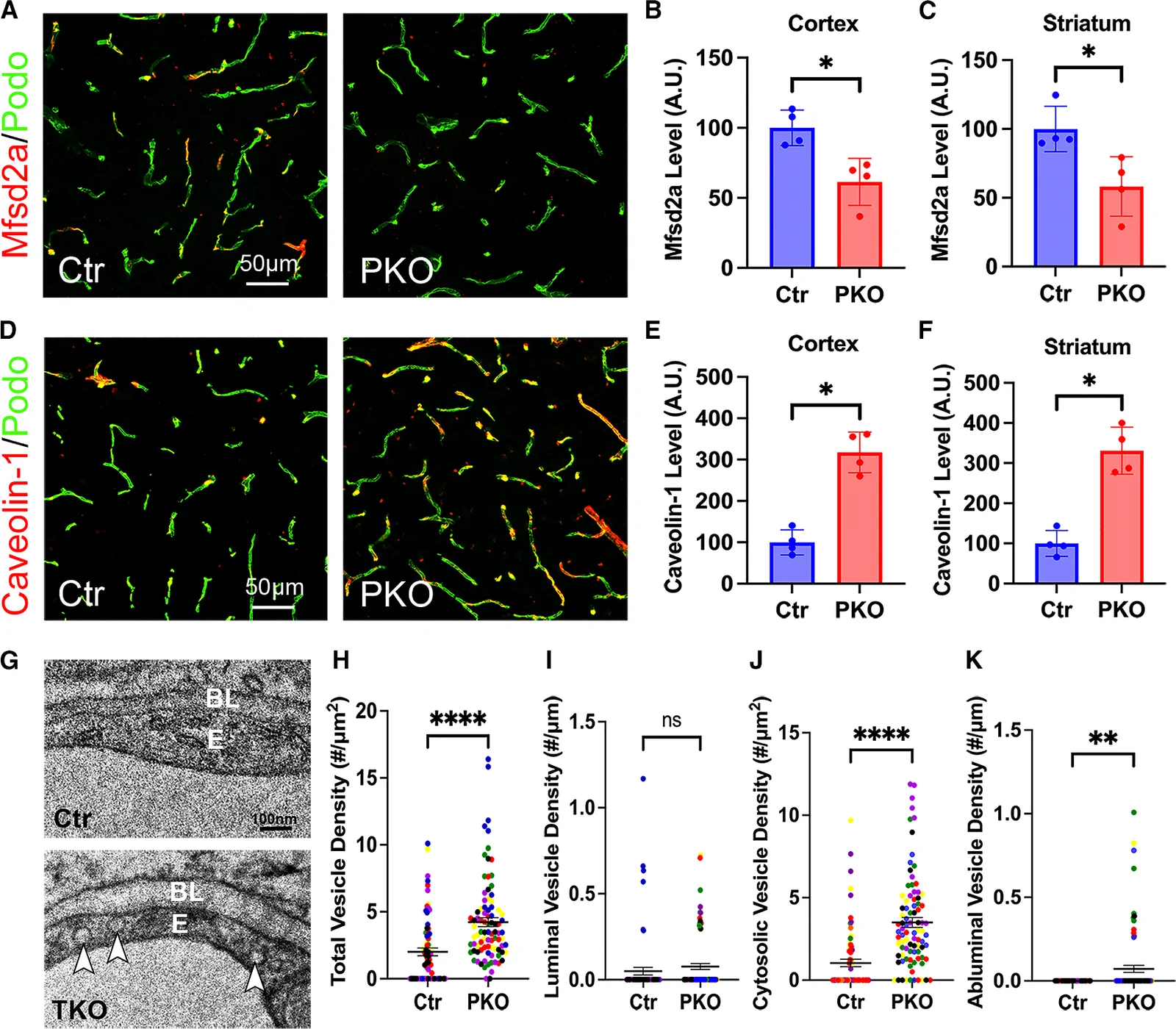
Transcellular transport is increased in PKO mice at 19–22 months (A) Immunohistochemistry showing Mfsd2a (red) and podocalyxin (green) staining in the cortex of 19- to 22-month-old control and PKO mice. Scale bars, 50 μm. (B) Quantifications of Mfsd2a levels in the cortex of 19- to 22-month-old control and PKO mice. *n* = 4 mice. *p = 0.0286, Mann-Whitney test. (C) Quantifications of Mfsd2a levels in the striatum of 19- to 22-month-old control and PKO mice. *n* = 4 mice. *p = 0.0286, Mann-Whitney test. (D) Immunohistochemistry showing caveolin-1 (red) and podocalyxin (green) staining in the cortex of 19- to 22-month-old control and PKO mice. Scale bars, 50 μm. (E) Quantifications of caveolin-1 levels in the cortex of 19- to 22-month-old control and PKO mice. *n* = 4 mice. *p = 0.0286, Mann-Whitney test. (F) Quantifications of caveolin-1 levels in the striatum of 19- to 22-month-old control and PKO mice. *n* = 4 mice. *p = 0.0286, Mann-Whitney test. (G) Representative TEM images showing endothelial pinocytotic vesicles (white arrowheads) in the brains of 19- to 22-month-old PKO mice. TEM, transmission electron microscopy; E, endothelium; BL, basal lamina. (H) Quantification of pinocytotic vesicle density in brain endothelial cells of control and PKO mice at 19–22 months. *n* = 73–88 capillaries from 6 mice. ****p <0.0001, unpaired Student’s *t* test. (I–K) Quantifications of endothelial pinocytotic vesicle density in the luminal membrane (I), cytosol (J), and abluminal membrane (K) in control and PKO mice at 19–22 months. *n* = 73–88 capillaries from 6 mice. **p = 0.0021, ****p <0.0001, unpaired Student’s *t* test. Data were shown as mean ± SD.

**Figure 4.**
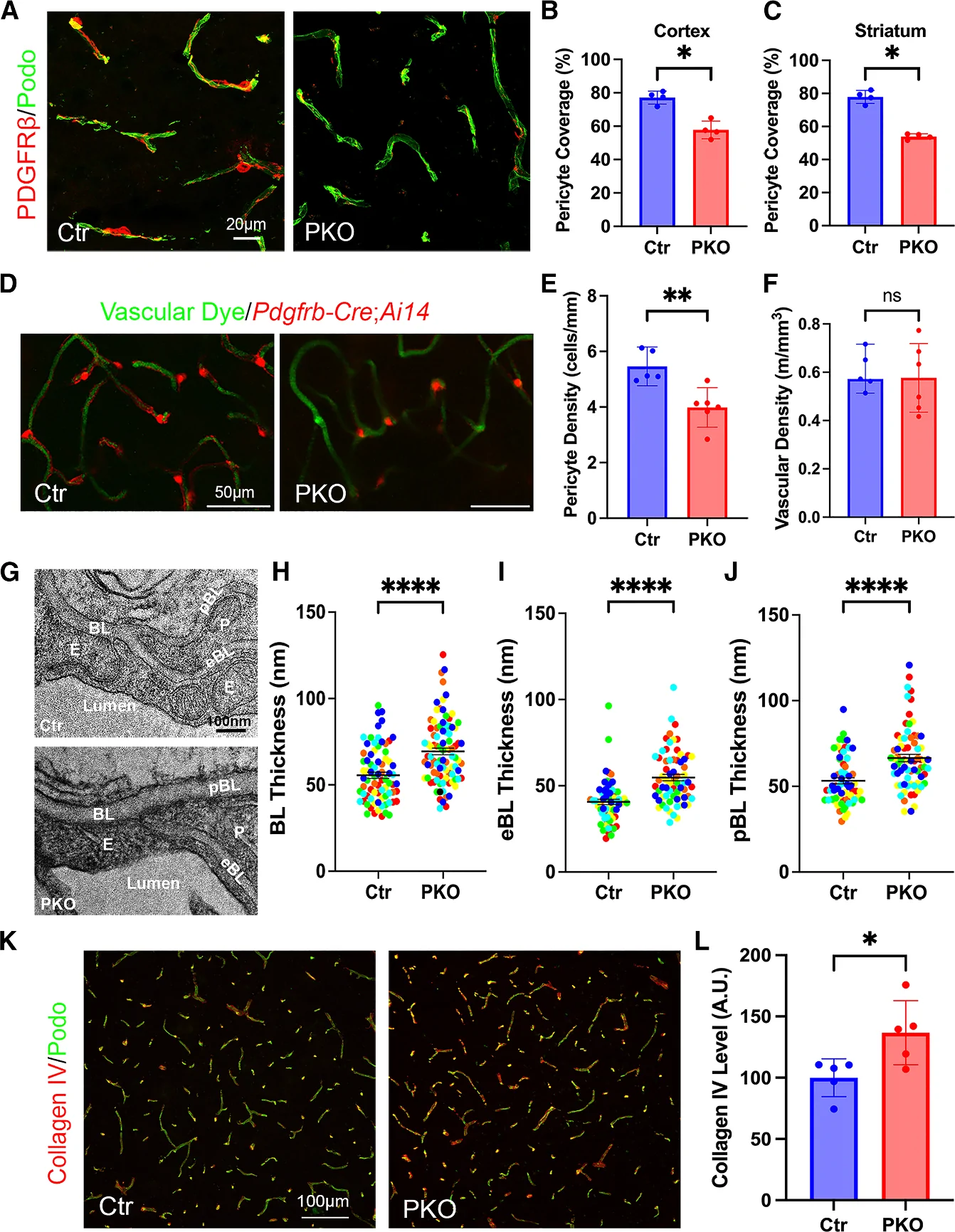
Pericyte loss is observed in PKO mice at 19–22 months (A) Immunohistochemistry showing PDGFRβ (red) and podocalyxin (green) staining in the cortex of 19- to 22-month-old control and PKO mice. Scale bars, 20 μm. (B) Quantification of PDGFRβ coverage in the cortex of 19- to 22-month-old control and PKO mice. *n* = 4 mice. *p = 0.0286, Mann-Whitney test. (C) Quantification of PDGFRβ coverage in the striatum of 19- to 22-month-old control and PKO mice. *n* = 4 mice. *p = 0.0286, Mann-Whitney test. (D) Representative two-photon image showing pericytes (red) and vasculature (green) in 19- to 22-month-old control and PKO mice. Scale bars, 50 μm. (E) Quantification of pericyte density (cells/mm of capillary length) in control (*n* = 5) and PKO mice (*n* = 6) at 19–22 months. **p = 0.0087, Mann-Whitney test. (F) Quantification of vascular density (m/mm^3^) in control (*n* = 5) and PKO mice (*n* = 6) at 19–22 months. ns, *p* = 0.6623, Mann-Whitney test. (G) Representative TEM images showing the BL, eBL, and pBL in the brains of 19- to 22-month-old control and PKO mice. Scale bars, 100 nm. TEM, transmission electron microscopy; E, endothelium; P, pericyte; BL, basal lamina; eBL, endothelial basal lamina; pBL, parenchymal basal lamina. (H) Quantification of BL thickness in the capillaries of 19- to 22-month-old control and PKO mice. *n* = 77–92 capillaries from 6 mice. ****p <0.0001, unpaired *t* test. (I) Quantification of eBL thickness in the capillaries of 19- to 22-month-old control and PKO mice. *n* = 64–68 capillaries from 6 mice. ****p <0.0001, unpaired *t* test. (J) Quantification of pBL thickness in the capillaries of 19- to 22-month-old control and PKO mice. *n* = 64–72 capillaries from 6 mice. ****p <0.0001, unpaired Student’s *t* test. (K) Immunohistochemistry showing collagen IV (red) and podocalyxin (green) staining in the cortex of 11- to 13-month-old control and PKO mice. Scale bars, 100 μm. (L) Quantification of collagen IV levels in the cortex of 11- to 13-month-old control and PKO mice. *n* = 5 mice. *p = 0.0329, unpaired Student’s *t* test. Data were shown as Mean ± SD.

**Figure 5.**
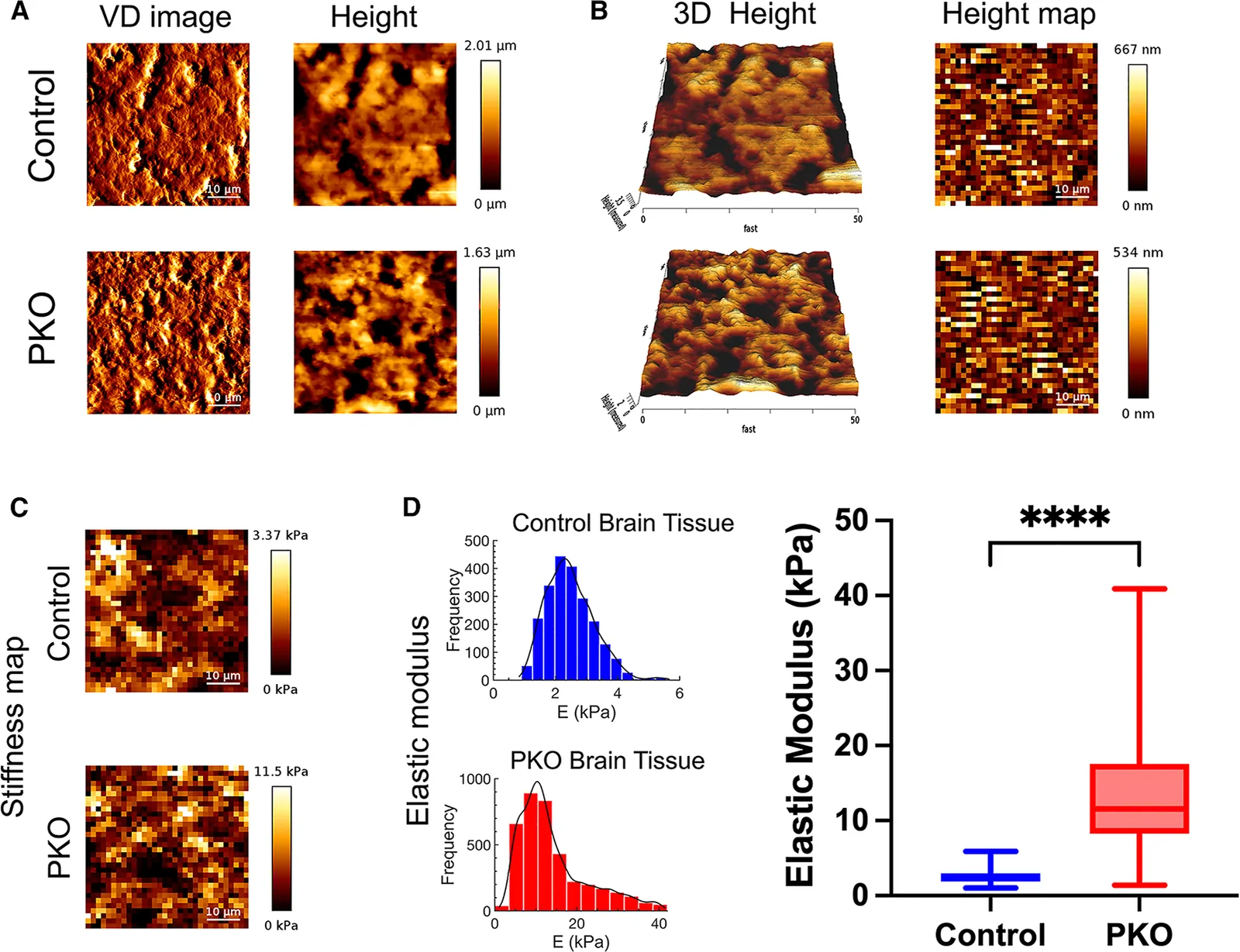
Brain stiffness is increased in PKO mice at 11–13 months (A) Representative AFM vertical deflection and height images obtained from selected brain regions of 11- to 13-month-old control and PKO mice (scan size: 50 μm × 50 μm). Distinct ultrastructural alterations are evident in PKO tissue relative to the control. AFM, atomic force microscopy. (B) Corresponding three-dimensional (3D) height renderings and height variation (standard deviation) maps highlighting enhanced local surface heterogeneity in 11- to 13-month-old PKO brains. (C) Spatially resolved maps of the elastic modulus showing color-coded distributions of local stiffness across representative regions of PKO and control brains at 11–13 months. (D) Histograms of elastic modulus values comparing the stiffness distributions between PKO and control brains at 11–13 months. Statistical analysis demonstrates significantly increased brain stiffness of PKO mice at 11–13 months compared to age-matched controls. *n* = 2233–3820 force curve measurements from 3 mice. ****p <0.0001, unpaired Student’s *t* test. Data were shown as boxplots.

**Figure 6.**
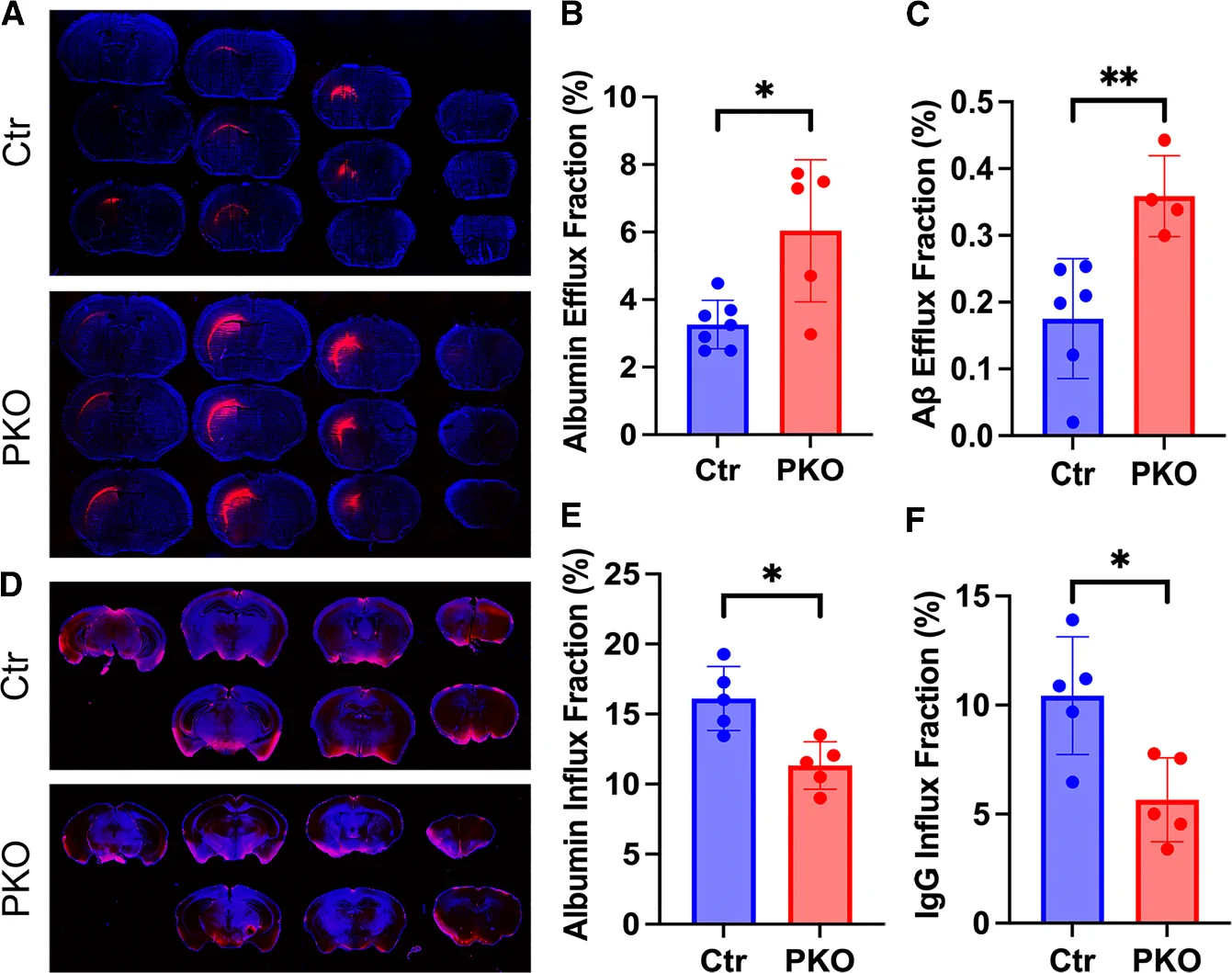
Brain efflux/influx functions are compromised in PKO mice at 19–22 months (A) Representative brain sections showing albumin-555 (red) coverage of the brain parenchyma in control and PKO mice at 19–22 months. (B) Quantification of albumin efflux fraction in control and PKO mice at 19–22 months. n = 5–7 mice. *p = 0.0303, Mann-Whitney test. (C) Quantification of Aβ efflux fraction in control and PKO mice at 19–22 months. n = 4–6 mice. **p = 0.0095, Mann-Whitney test. (D) Representative brain sections showing albumin-555 (red) influx into the brain parenchyma of control and PKO mice at 19–22 months. (E) Quantification of albumin influx fraction in control and PKO mice at 19–22 months. *n* = 5 mice. *p = 0.0159, Mann-Whitney test. (F) Quantification of IgG influx fraction in control and PKO mice at 19–22 months. *n* = 5 mice. *p = 0.0317, Mann-Whitney test. Data were shown as mean ± SD.

**Figure 7.**
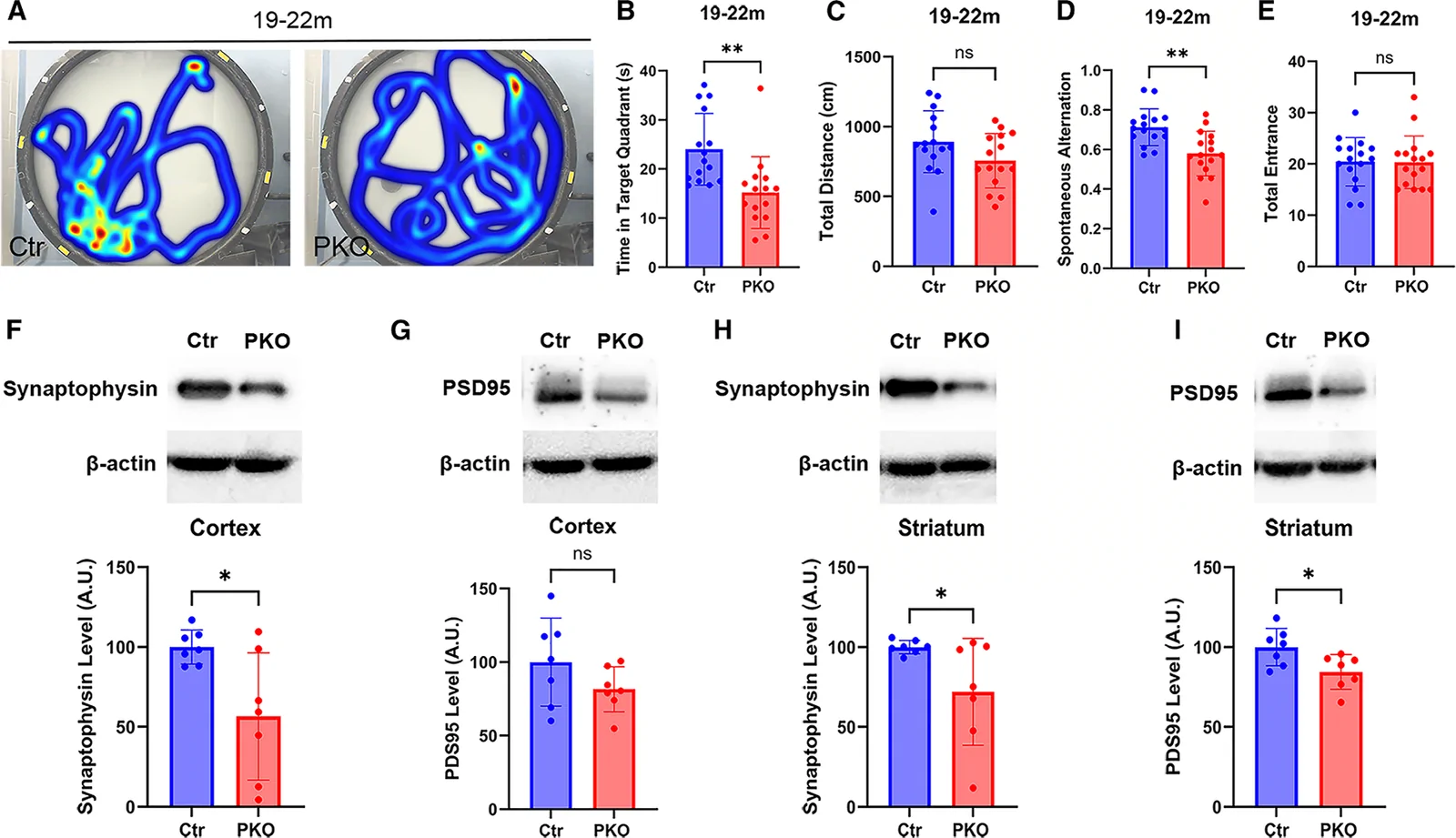
Cognitive function is impaired in PKO mice at 19–22 months (A) Representative track plots for 19- to 22-month-old control and PKO mice in the probe trial of the Morris water maze test. The lower left quadrant is the target quadrant. (B) Quantification showing that 19- to 22-month-old PKO mice spend less time in the target quadrant in the Morris water maze test compared to age-matched controls. *n* = 15 mice. **p = 0.0025, unpaired Student’s *t* test. (C) Quantification revealing comparable total swimming distance in 19- to 22-month-old control and PKO mice in the Morris water maze test. *n* = 15 mice. ns, *p* = 0.0841, unpaired Student’s *t* test. (D) Quantification revealing decreased spontaneous alternation of 19- to 22-month-old PKO mice in the Y maze test compared to age-matched controls. *n* = 16 mice. **p = 0.001, unpaired Student’s *t* test. (E) Quantification revealing comparable total arm entrance of 19- to 22-month-old PKO mice in the Y maze test compared to age-matched controls. *n* = 16 mice. ns, *p* = 0.9432, unpaired Student’s *t* test. (F) Western blotting and quantification of synaptophysin levels in the cortex of 19- to 22-month-old control and PKO mice. *n* = 7 mice. *p = 0.0164, unpaired Student’s *t* test. (G) Western blotting and quantification of PSD95 levels in the cortex of 19- to 22-month-old control and PKO mice. *n* = 7 mice. ns, *p* = 0.173, unpaired Student’s *t* test. (H) Western blotting and quantification of synaptophysin levels in the striatum of 19- to 22-month-old control and PKO mice. *n* = 7 mice. *p = 0.0483, unpaired Student’s *t* test. (I) Western blotting and quantification of PSD95 levels in the striatum of 19- to 22-month-old control and PKO mice. *n* = 7 mice. *p = 0.0245, unpaired Student’s *t* test. Data were shown as mean ± SD.

**KEY RESOURCES TABLE:** 

REAGENT or RESOURCE	SOURCE	IDENTIFIER

Antibodies		

Rabbit anti-hemoglobin	Cloud-Clone	Cat#PAB409Mu01
Goat anti-albumin	Novus Biologicals	Cat#NB600-41532; RRID:AB_2258189
Goat anti-podocalyxin	R&D	Cat#AF1556; RRID:AB_354858
Rabbit anti-Mfsd2a	Dr. Chenghua Gu Lab	N/A
Mouse anti-caveolin-1	Santa Cruz	Cat#sc-70516; RRID:AB_1120056
Rabbit anti-ZO1	Thermo Fisher Scientific	Cat#61-7300; RRID:AB_2533938
Mouse anti-claudin5	Thermo Fisher Scientific	Cat#35-2500; RRID:AB_2533200
Rat anti-CD31	BD	Cat#553370; RRID:AB_394816
Rabbit anti-collagen IV	Abcam	Cat#ab6586; RRID:AB_305584
Rat anti-nidogen1	Thermo Fisher Scientific	Cat#MA1-06501; RRID:AB_558792
Rabbit anti-GFAP	Thermo Fisher Scientific	Cat#PA1-10019; RRID:AB_1074611
Rat anti-PDGFRβ	eBioscience	Cat#AF2535; RRID:AB_2063012
Rabbit anti-PSD95	Abcam	Cat#ab18258; RRID:AB_444362
Mouse anti-synaptophysin	Santa Cruz	Cat#sc-12737; RRID:AB_628313
Mouse anti-β-actin	BD	Cat#612657; RRID:AB_399901
Alexa Fluor 594 conjugated donkey anti-rabbit	Thermo Fisher Scientific	Cat#A21207; RRID:AB_141637
Alexa Fluor 555 conjugated donkey anti-mouse	Thermo Fisher Scientific	Cat#A31570; RRID:AB_2536180
Alexa Fluor 488 conjugated donkey anti-goat	Thermo Fisher Scientific	Cat#A11055; RRID:AB_2534102
Alexa Fluor 555 conjugated donkey anti-goat	Thermo Fisher Scientific	Cat#A21432; RRID:AB_2535853
Alexa Fluor 647 conjugated donkey anti-goat	Thermo Fisher Scientific	Cat#A21447; RRID:AB_141844
Alexa Fluor 488 conjugated donkey anti-rat	Thermo Fisher Scientific	Cat#A21208; RRID:AB_2535794
Alexa Fluor 555 conjugated donkey anti-rat	Thermo Fisher Scientific	Cat#A78945; RRID:AB_2910652
HRP conjugated donkey anti-rabbit	Southern Biotech	Cat#64400; RRID:AB_619906
HRP conjugated donkey anti-mouse	Southern Biotech	Cat#A641505; RRID:AB_2796327

Chemicals, peptides, and recombinant proteins		

HiLyte Fluor 647-labeled Aβ1-42	AnaSpec	Cat#AS64161
Alexa Fluor 555-conjugated albumin	Thermo Fisher Scientific	Cat#A34786
FITC conjugated dextran (4 kDa)	Sigma	Cat#FD4
FITC conjugated dextran (2 MDa)	Sigma	Cat#FD2000S

Critical commercial assays		

M.O.M.^™^ Kit	Vector Laboratories, Inc.	Cat#FMK-2201
BCA Protein Assay Kit	Pierce	Cat#23227
Clarity Max^™^ Western ECL Substrate	Bio-Rad	Cat#1705062

Deposited Data		

Raw data	This paper	http://www.doi.org/10/17632/t8hszf89km.2

Experimental models: Organisms/strains		

Mouse: B6.129P2-*Lamc1^tm1Strl^*/J	The Jackson Laboratory	JAX: 016917
Mouse: B6.Cg-Gt(ROSA) *26Sor^tm14(CAG-tdTomato)Hze^*/J	The Jackson Laboratory	JAX: 007914
Mouse: Pdgfrb-Cre	Dr. Volkhard LindnerH	N/A
Mouse: B6.FVB-Tg(Col1a1-cre)1Kry	RIKEN BioResource Research Center	RIKEN: RBRC05603

Software and algorithms		

ImageJ	NIH	https://imagej.nih.gov/ij/
GraphPad Prism 9	GraphPad	https://www.graphpad.com/features
Photoshop	Adobe	https://www.adobe.com

## Highlights

- Loss of pericytic laminin-γ1 induces BBB disruption in an age-dependent manner
- Loss of pericytic laminin-γ1 leads to pericyte loss, BL thickening, and brain stiffening
- Loss of pericytic laminin-γ1 compromises CSF influx/brain clearance at old age
- Loss of pericytic laminin-γ1 results in synaptic loss and cognitive impairment at old age
