# Supplementary material for: Loss of laminin-γ1 in pericytes impairs microvascular function and cognition
Source: Cell Rep. Author manuscript; Available in PMC 2026 Oct 12. (PMC13657255; doi:10.1016/j.celrep.2026.117904)
Supplement: Supplementary file 1 [file NIHMS2212225-supplement-1.pdf]

Cell Reports, Volume *45*

Supplemental information

Loss of laminin-γ1 in pericytes impairs microvascular function and cognition

Jingsong Ruan, Ava Nasrollahi, Cecilia Martin, Meisam Asgari, Andy Y. Shih, and Yao Yao

Supplemental Figures

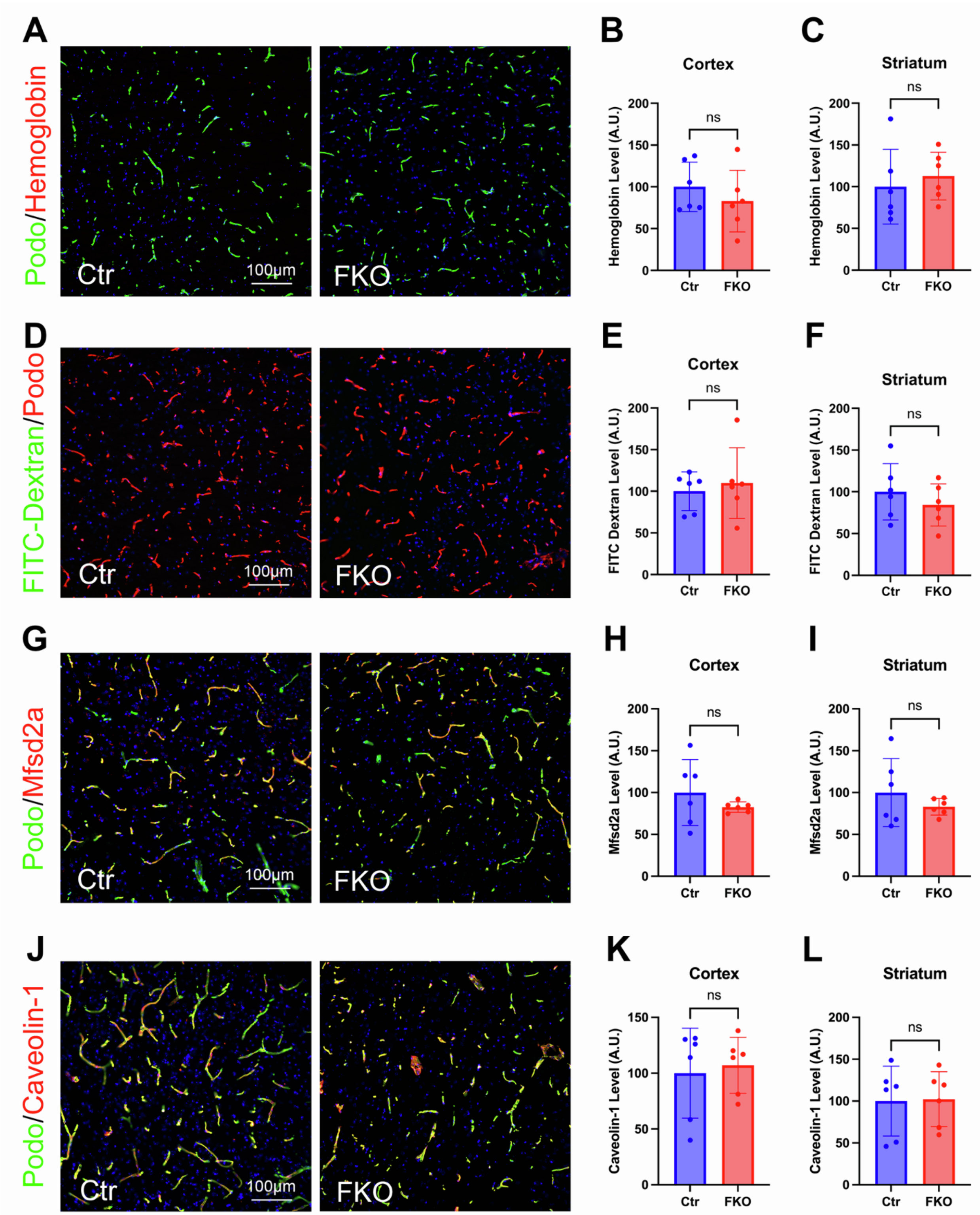

Figure S1. BBB integrity is intact in 19-month-old FKO mice.

(A) Immunohistochemistry showing hemoglobin (red) and podocalyxin (green) staining in the cortex of 19-month-old control and FKO mice. Scale bar, 100μm. FKO, *Lamc1*$^{flox/flox}$:*Col1a1*-Cre^{+}.

(B) Quantification of hemoglobin leakage in the cortex of 19-month-old control and FKO mice. n = 6 mice. ns, p = 0.6991, Mann-Whitney Test.

(C) Quantification of hemoglobin leakage in the striatum of 19-month-old control and FKO mice. n = 6 mice. ns, p = 0.3095, Mann-Whitney Test.

(D) Immunohistochemistry showing 4kD-FITC-Dextran (green) and podocalyxin (red) staining in the cortex of 19-month-old control and FKO mice. Scale bar, 100μm.

(E) Quantification of 4kD-FITC-Dextran leakage in the cortex of 19-month-old control and FKO mice. n = 6 mice. ns, p = 0.6991, Mann-Whitney Test.

(F) Quantification of 4kD-FITC-Dextran leakage in the striatum of 19-month-old control and FKO mice. n = 6 mice. ns, p = 0.5887, Mann-Whitney Test.

(G) Immunohistochemistry showing Mfsd2a (red) and podocalyxin (green) staining in the cortex of 19-month-old control and FKO mice. Scale bar, 100μm.

(H) Quantification of Mfsd2a expression in the cortex of 19-month-old control and FKO mice. n = 6 mice. ns, p = 0.4848, Mann-Whitney Test.

(I) Quantification of Mfsd2a expression in the striatum of 19-month-old control and FKO mice. n = 6 mice. ns, p = 0.9372, Mann-Whitney Test.

(J) Immunohistochemistry showing caveolin-1 (red) and podocalyxin (green) staining in the cortex of 19-month-old control and FKO mice. Scale bar, 100μm.

(K) Quantification of caveolin-1 expression in the cortex of 19-month-old control and FKO mice. n = 6 mice. ns, $p > 0.9999$, Mann-Whitney Test.

(L) Quantification of caveolin-1 expression in the striatum of 19-month-old control and FKO mice. n = 6 mice. ns, p = 0.8182, Mann-Whitney Test.

Data were shown as Mean ± SD. Related to Figure 1.

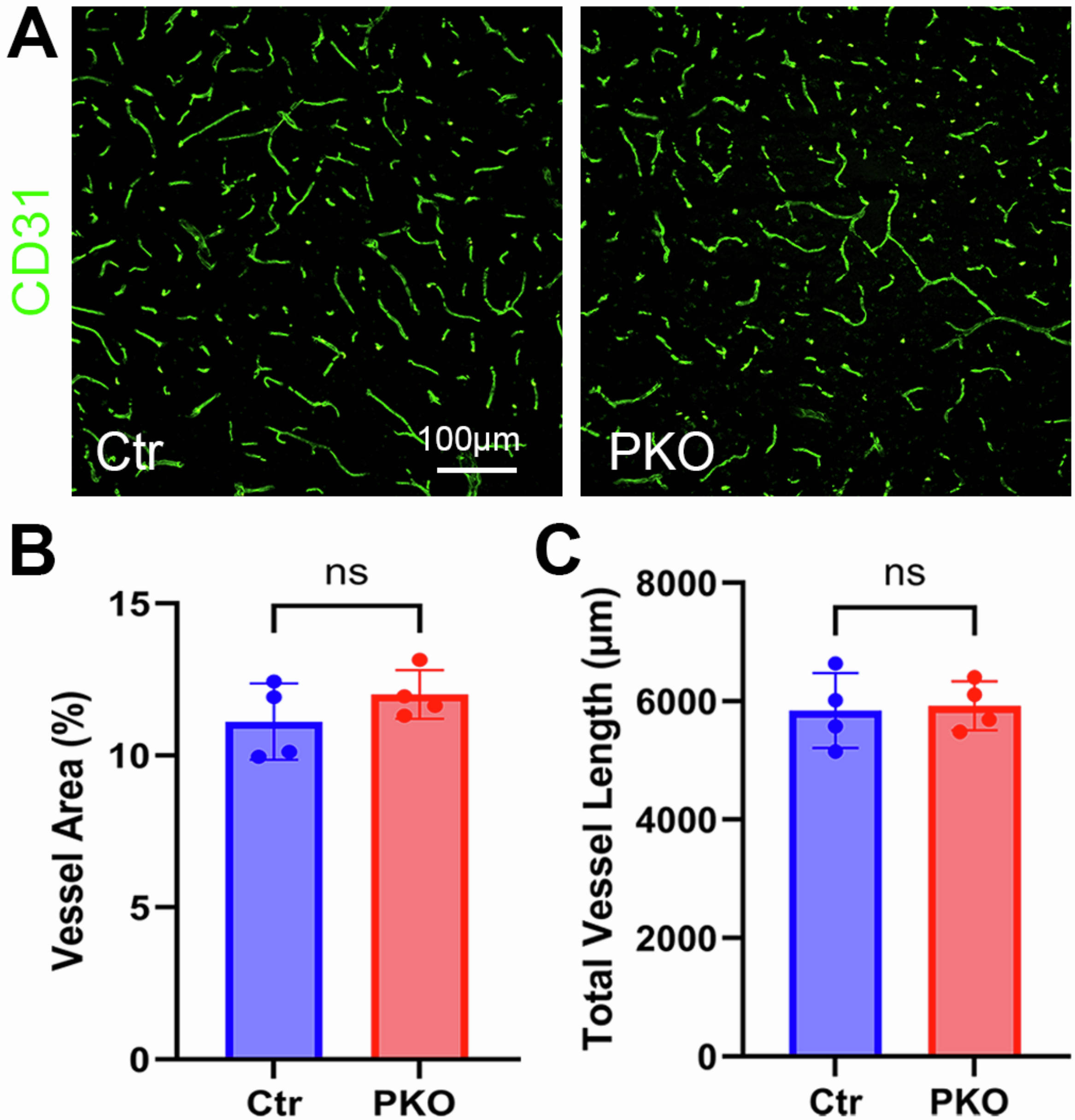
A
CD31
Ctr
100μm
PKO
B
ns
Vessel Area (%)
15
10
5
0
Ctr
PKO
C
ns
Total Vessel Length (μm)
8000
6000
4000
2000
0
Ctr
PKO

Figure S2. Vessel density is unaffected in PKO mice at 11-13 months.
(A) Immunohistochemistry showing CD31 (green) staining in the cortex of control and PKO mice at 19-22 months. Scale bar, 100µm.
(B) Quantification of vessel area (%) in the cortex of 19- to 22-month-old control and PKO mice. $n = 4$ mice. ns, $p = 0.4857$, Mann-Whitney Test.
(C) Quantification of total vessel length in the cortex of 19- to 22-month-old control and PKO mice. $n = 4$ mice. ns, $p = 0.8857$, Mann-Whitney Test.
Data were shown as Mean ± SD. Related to Figure 4.

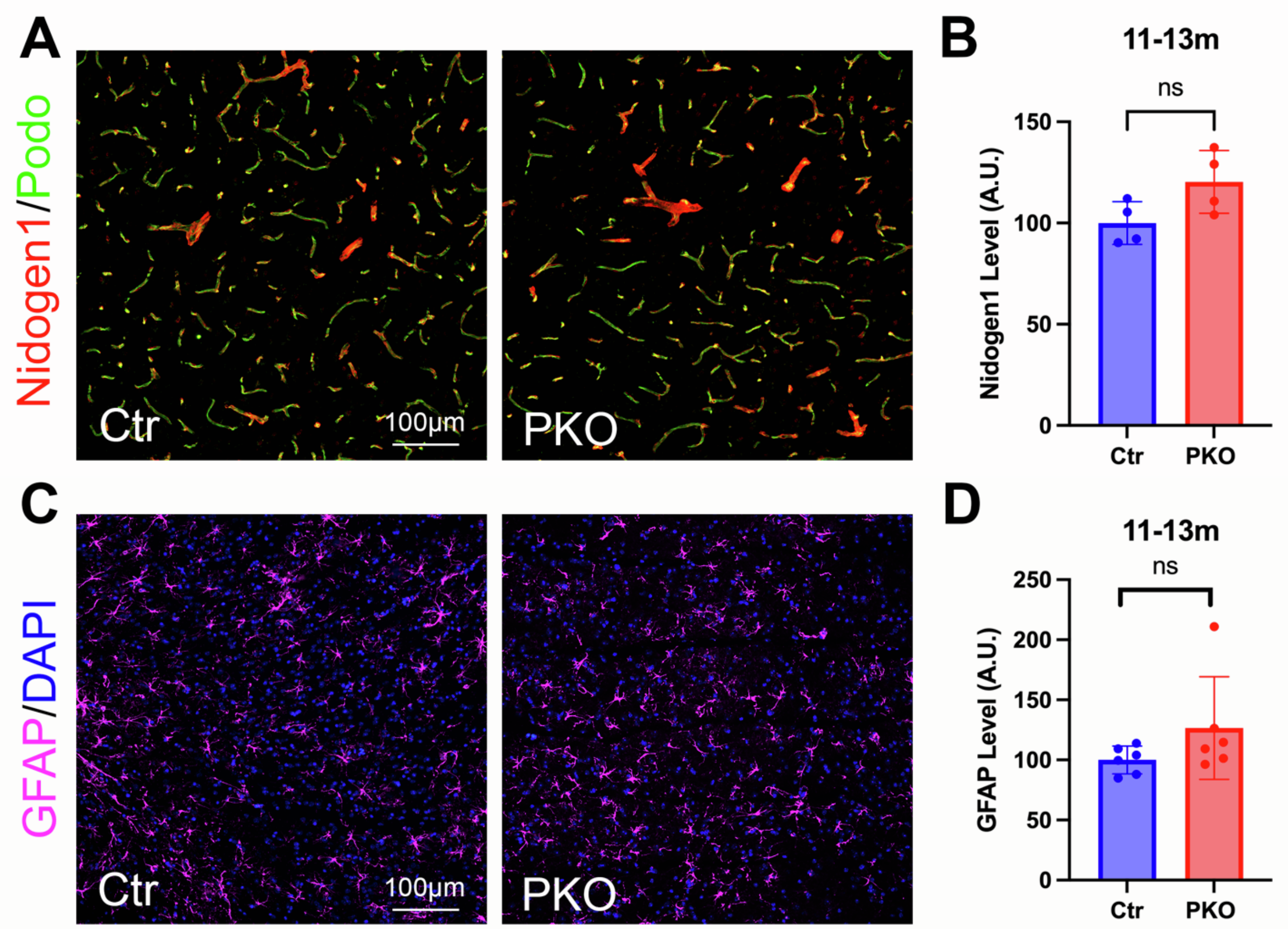

A
Nidogen1/Podo
Ctr
100μm
PKO
B
11-13m
ns
Nidogen1 Level (A.U.)
150
100
50
0
Ctr
PKO
C
GFAP/DAPI
Ctr
100μm
PKO
D
11-13m
ns
GFAP Level (A.U.)
250
200
150
100
50
0
Ctr
PKO

Figure S3. Nidogen1 and GFAP expression in PKO mice at 11-13 months.
(A) Immunohistochemistry showing nidogen1 (red) and podocalyxin (green) staining in the cortex of 11- to 13-month-old control and PKO mice. Scale bar, 100μm.
(B) Quantification of nidogen1 levels in the cortex of 11- to 13-month-old control and PKO mice. n = 4 mice. ns, p = 0.2000, Mann-Whitney Test.
(C) Immunohistochemistry showing GFAP (magenta) staining in the cortex of 11- to 13-month-old control and PKO mice. Scale bar, 100μm.
(D) Quantification of GFAP levels in the cortex of 11- to 13-month-old control and PKO mice. n = 6 mice. ns, p = 0.1797, Mann-Whitney Test.
Data were shown as Mean ± SD. Related to Figure 4.

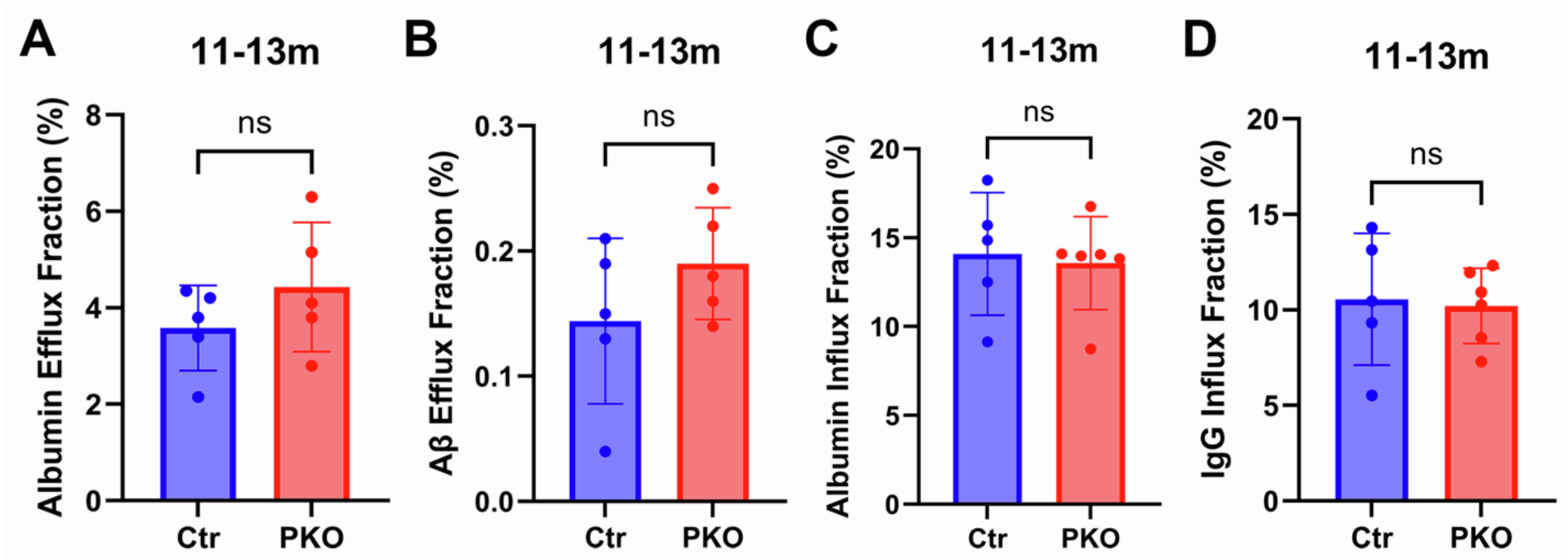
A
11-13m
Albumin Efflux Fraction (%)
8
6
4
2
0
ns
Ctr
PKO
B
11-13m
Aβ Efflux Fraction (%)
0.3
0.2
0.1
0.0
ns
Ctr
PKO
C
11-13m
Albumin Influx Fraction (%)
20
15
10
5
0
ns
Ctr
PKO
D
11-13m
IgG Influx Fraction (%)
20
15
10
5
0
ns
Ctr
PKO

Figure S4. Brain efflux/influx functions are unaffected in PKO mice at 11-13 months.
(A) Quantification of Albumin efflux fraction in 11- to 13-month-old control and PKO mice. *n* = 5 mice. ns, $p = 0.4603$, Mann-Whitney Test.
(B) Quantification of Aβ efflux fraction in 11- to 13-month-old control and PKO mice. *n* = 5 mice. ns, $p = 0.3095$, Mann-Whitney Test.
(C) Quantification of Albumin influx fraction in 11- to 13-month-old control and PKO mice. *n* = 5-6 mice. ns, $p = 0.6623$, Mann-Whitney Test.
(D) Quantification of IgG influx fraction in 11- to 13-month-old control and PKO mice. *n* = 5-6 mice. ns, $p = 0.7922$, Mann-Whitney Test.
Data were shown as Mean ± SD. Related to Figure 6.

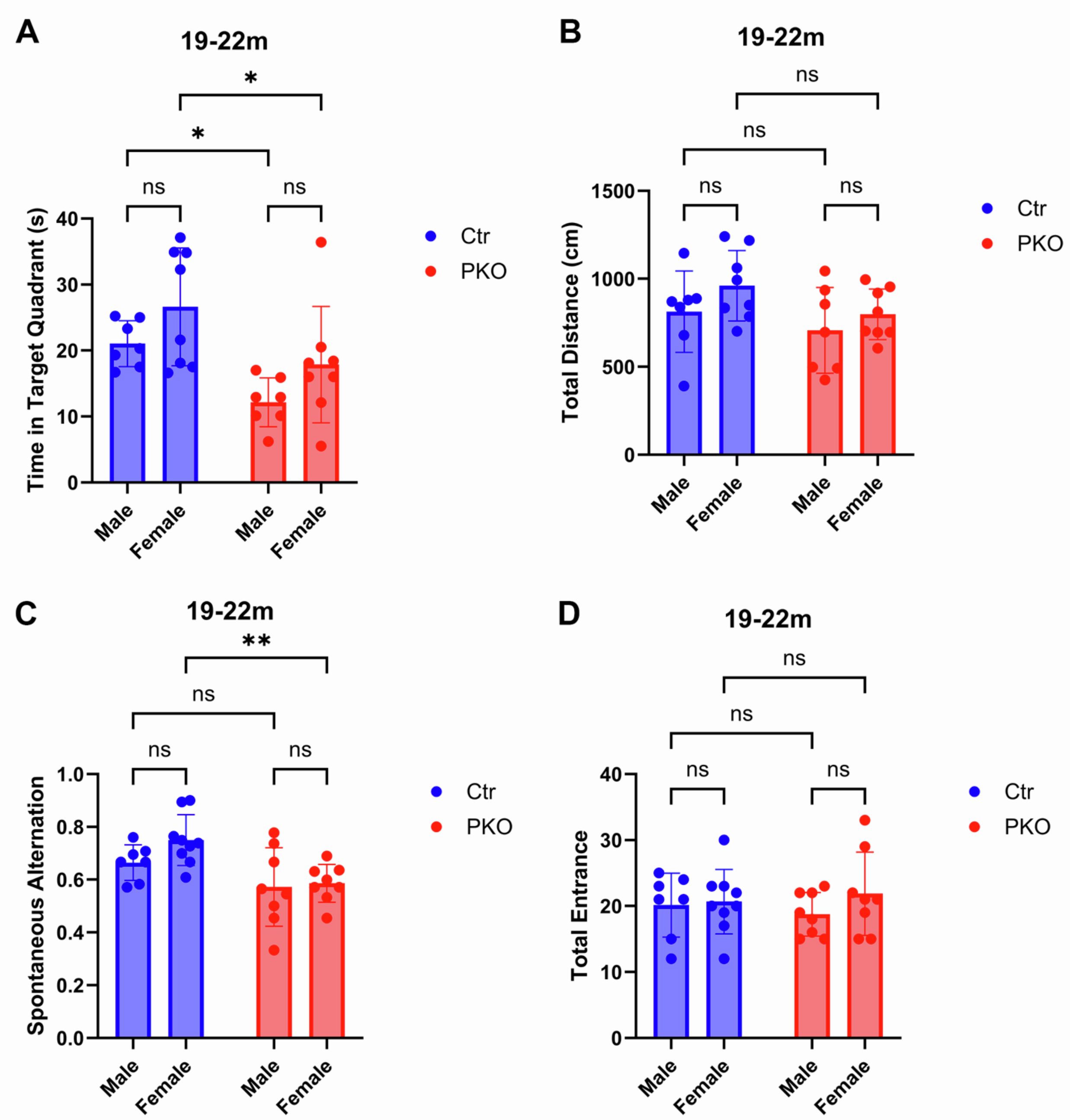
A
19-22m
Time in Target Quadrant (s)
Male
Female
Male
Female
ns
ns
*
*
Ctr
PKO
B
19-22m
Total Distance (cm)
ns
ns
ns
ns
Ctr
PKO
C
19-22m
Spontaneous Alternation
ns
ns
ns
**
Ctr
PKO
D
19-22m
Total Entrance
ns
ns
ns
ns
Ctr
PKO

Figure S5. Sex-specific alterations of cognitive function in PKO mice at 19-22 months.
(A) Quantification showing that both male and female PKO mice at 19-22 months spend less time in the target quadrant in the Morris water maze test compared to the controls. n = 7-8 mice. ns, p = 0.1340 for control males and control females; ns, p = 0.1244 for PKO males and PKO females; *p = 0.0244 for control males and PKO males; *p = 0.0186 for control females and PKO females; Two-way ANOVA.
(B) Quantification revealing comparable total swimming distance in male and female mice of both genotypes (control and PKO) in the Morris water maze test. n = 7-8 mice. ns, p = 0.5363 for control males and control females; ns, p = 0.8712 for PKO males and PKO females; ns, p = 0.8263 for control males and PKO males; ns, p = 0.4174 for control females and PKO females; Two-way ANOVA.
(C) Quantification revealing decreased spontaneous alternation of female but not male PKO mice at 19-22 months in the Y maze test compared to the controls. n = 7-9 mice. ns, p = 0.1077 for control males and control females; ns, p = 0.7925 for PKO males and PKO females; ns, p = 0.0925 for control males and PKO males; ns, **p = 0.0026 for control females and PKO females; Two-way ANOVA.
(D) Quantification revealing comparable total arm entrance in male and female mice of both genotypes (control and PKO) in the Morris water maze test. n = 7-8 mice. ns, p = 0.9258 for control males and control females; ns, p = 0.1818 for PKO males and PKO females; ns, p = 0.5119 for control males and PKO males; ns, p = 0.6202 for control females and PKO females; Two-way ANOVA.
Data were shown as Mean ± SD. Related to Figure 7.

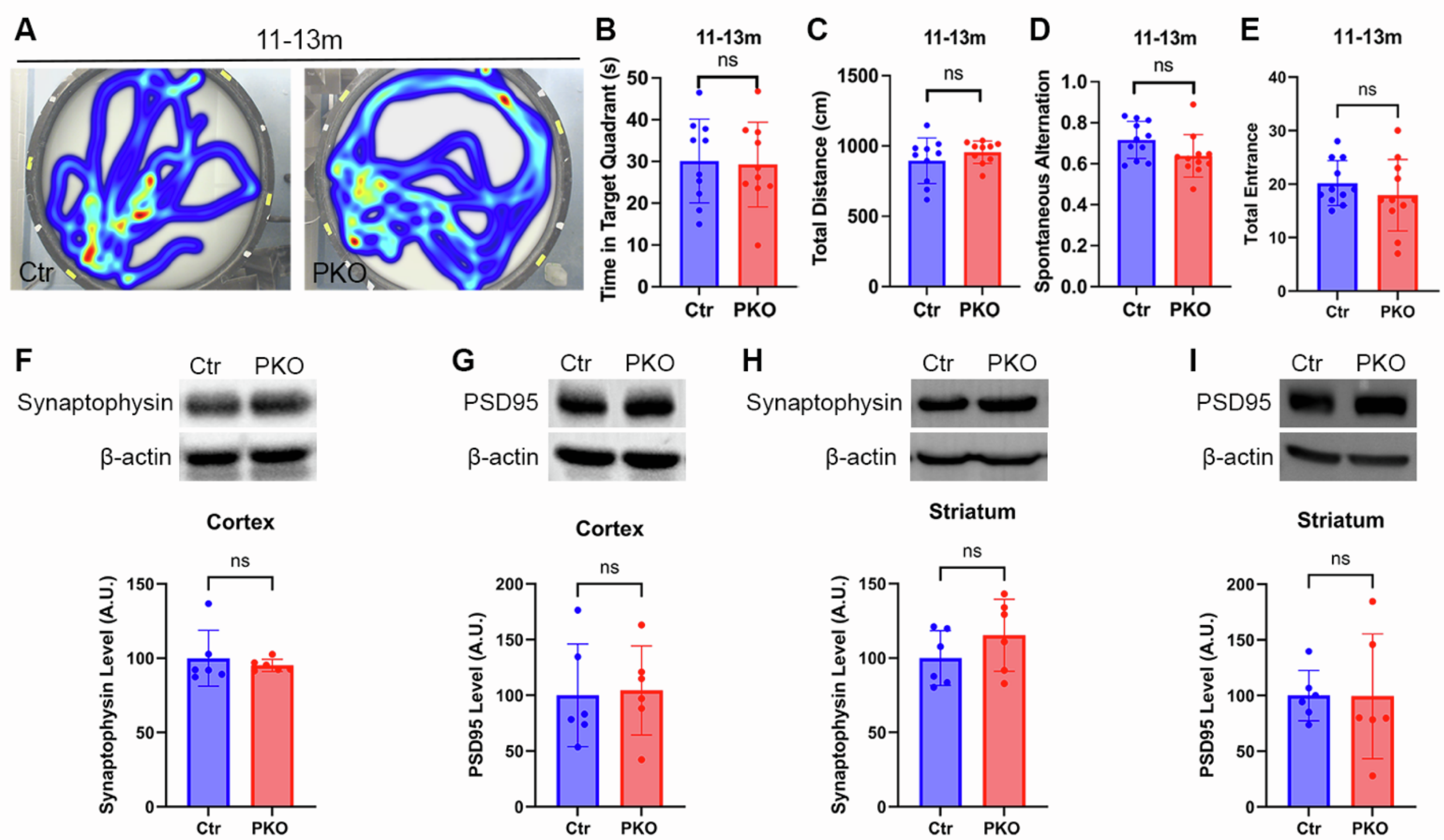

A
11-13m
Ctr
PKO
B
11-13m
Time in Target Quadrant (s)
ns
C
11-13m
Total Distance (cm)
ns
D
11-13m
Spontaneous Alternation
ns
E
11-13m
Total Entrance
ns
F
Synaptophysin
β-actin
Cortex
Synaptophysin Level (A.U.)
G
PSD95
β-actin
Cortex
PSD95 Level (A.U.)
H
Synaptophysin
β-actin
Striatum
Synaptophysin Level (A.U.)
I
PSD95
β-actin
Striatum
PSD95 Level (A.U.)

Figure S6. Cognitive function is unaffected in PKO mice at 11-13 months.
(A) Representative track plots for 11- to 13-month-old control and PKO mice in the probe trial of the Morris water maze test. The lower left quadrant is the target quadrant.
(B) Quantification showing that 11- to 13-month-old PKO mice spend less time in the target quadrant in the Morris water maze test compared to age-matched controls. n = 10 mice. ns, p = 0.8613, Unpaired Student's t Test.
(C) Quantification revealing comparable total swimming distance in 11- to 13-month-old control and PKO mice in the Morris water maze test. n = 10 mice. ns, p = 0.3128, Unpaired Student's t Test.
(D) Quantification revealing decreased spontaneous alternation of 11- to 13-month-old PKO mice in the Y maze test compared to age-matched controls. n = 11 mice. ns, p = 0.0753, Unpaired Student's t Test.
(E) Quantification revealing comparable total arm entrance of 11- to 13-month-old PKO mice in the Y maze test compared to age-matched controls. n = 11 mice. ns, p = 0.3529, Unpaired Student's t Test.
(F) Western blotting and quantification of synaptophysin levels in the cortex of 11- to 13-month-old control and PKO mice. n = 6 mice. ns, p = 0.5643, Unpaired Student's t Test.
(G) Western blotting and quantification of PSD95 levels in the cortex of 11- to 13-month-old control and PKO mice. n = 6 mice. ns, p = 0.8636, Unpaired Student's t Test.
(H) Western blotting and quantification of synaptophysin levels in the striatum of 11- to 13-month-old control and PKO mice. n = 6 mice. ns, p = 0.2499, Unpaired Student's t Test.
(I) Western blotting and quantification of PSD95 levels in the striatum of 11- to 13-month-old control and PKO mice. n = 6 mice. ns, p = 0.9813, Unpaired Student's t Test.
Data were shown as Mean ± SD. Related to Figure 7.

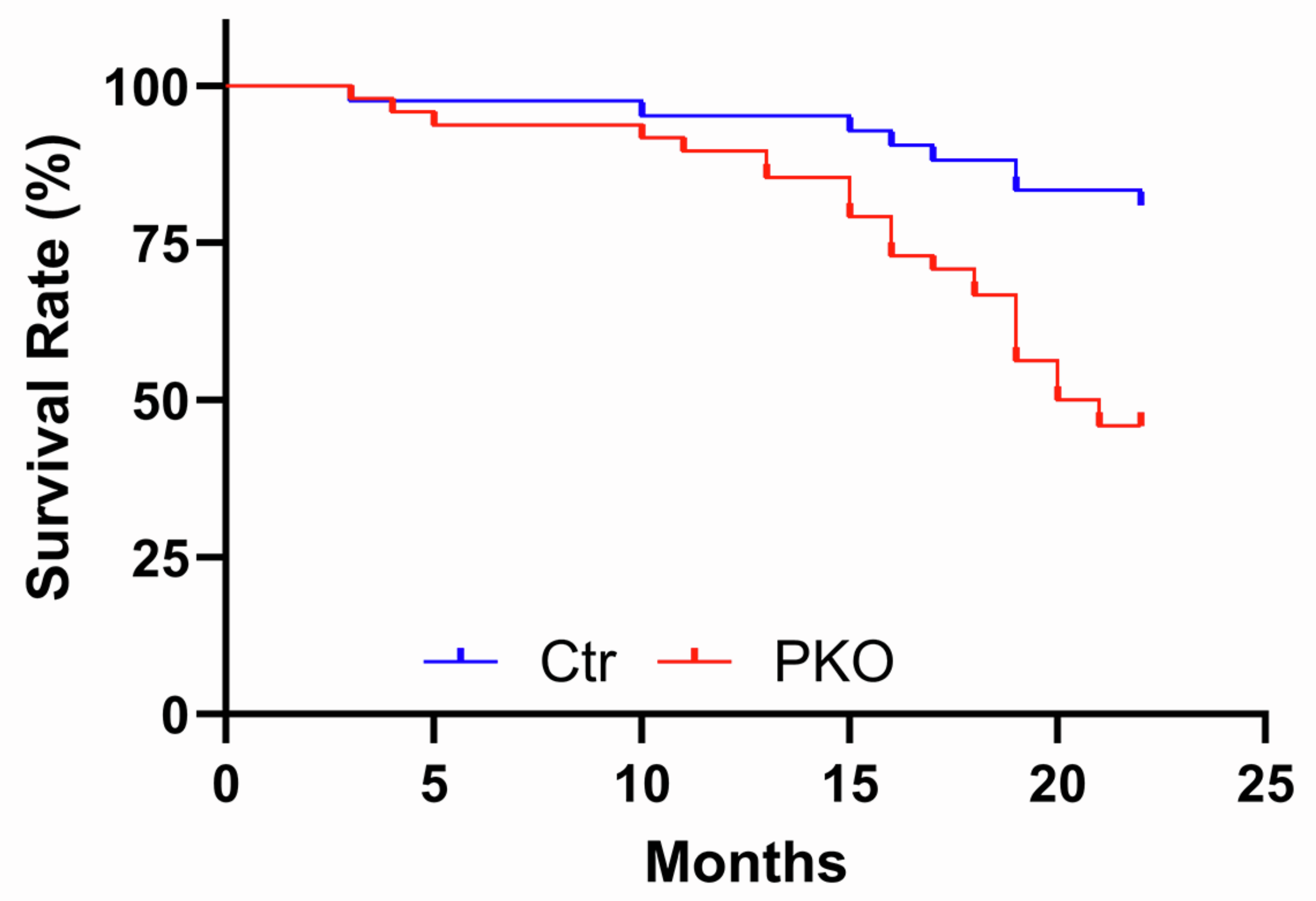

Survival Rate (%)
100
75
50
25
0
0
5
10
15
20
25
Months
Ctr
PKO

Figure S7. PKO mice exhibit reduced survival rate.
Survival curve showing reduced survival rate of PKO mice at 15-22 months. n = 42-48. ***p = 0.0008, Log-rank Test.
Related to Figure 7.
